# AntLink: A Biomimicry-Orientated System for Workflow Improvement at an Automobile Aftersales Service Centre

**DOI:** 10.3390/biomimetics11100756

**Published:** 2026-10-19

**Authors:** Carla Du Plessis, Meelan Roopa, Edward Davies

**Affiliations:** Department of Industrial Engineering, Stellenbosch University, Stellenbosch 7602, South Africa; 26353903@sun.ac.za (C.D.P.); 18282660@sun.ac.za (E.D.)

**Keywords:** biomimicry, nature-inspired design, automobile aftersales services, Analytic Hierarchy Process, root cause analysis, decentralised systems, sustainable waste management

## Abstract

The South African automotive industry is under increasing pressure to improve the operational efficiency and sustainability of its aftersales services. Biomimicry was applied to enhance maintenance, repair and waste management processes at a medium-sized Western Cape dealership. Although overall customer satisfaction scores were high, significant gaps were identified between customer treatment ratings and satisfaction with repair quality, indicating inefficiencies in service delivery. Repeat customer complaints persisted due to diagnostic errors, inconsistent repair quality, and lack of innovative sustainable waste practices. The Biomimicry Challenge to Biology methodology guided the research. Root cause analysis tools (Business Process Mapping, Why-Why trees, and Fishbone Diagrams) identified seven functional requirements, mapped to the Biomimicry Taxonomy to derive nature-inspired design principles. Three biomimicry concepts were generated and evaluated using the Analytic Hierarchy Process; the highest-ranking concept, Decentralised Resource Distribution Inspired by Ant Colonies, was emulated into AntLink, a digital system that replaces rigid hierarchy with a collaborative, real-time “digital pheromone trail” linking managers, service advisors and technicians. Verification confirmed that AntLink satisfied all functional requirements, including first-time repair resolution targets, standardised diagnostic checklists for recurring processes, and waste category monitoring. Validation combined stakeholder surveys across operational roles, showing overall positive consensus with good agreement on functional requirement alignment, and an assessment against Biomimicry 3.8’s Life’s Principles confirmed conceptual alignment with resilience, adaptability and sustainability. AntLink illustrates a practical route for translating nature-inspired design into measurable operational and environmental gains in the automotive aftersales sector.

## 1. Introduction

**Figure 1:**
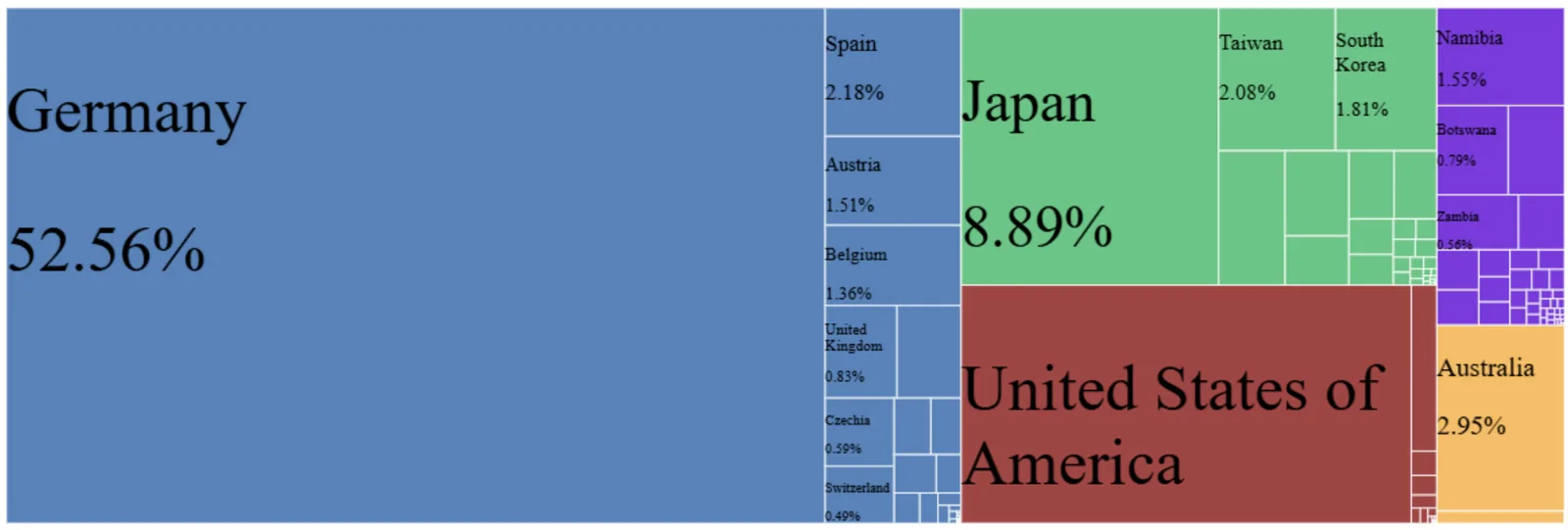
Locations South Africa exported cars to in 2022, generated with Growth Lab (The Atlas of Economic Complexity, 2022).

The South African automotive industry is one of the country’s largest economic sectors, generating 4.3% of national GDP, contributing over 17% of national manufacturing production [1]. Beyond domestic sales, the sector is a substantial contributor to South Africa’s international trade; Figure 1 illustrates the country’s principal vehicle export destinations in 2022. Automobile aftersales services represent a particularly profitable opportunity within this sector, allowing dealerships to capitalise on customers’ ongoing needs and establish a recurring revenue stream [2]. The aftersales industry, classified under ISIC Code 4520 (maintenance and repair of motor vehicles), covers the servicing, repair, maintenance and disposal activities that follow a vehicle purchase. Customer expectations in this space are evolving: growing sustainability demands mean customers increasingly seek green maintenance practices and responsible waste management programmes [3], and meeting these needs improves both customer satisfaction and environmental outcomes.

**Figure 2:**
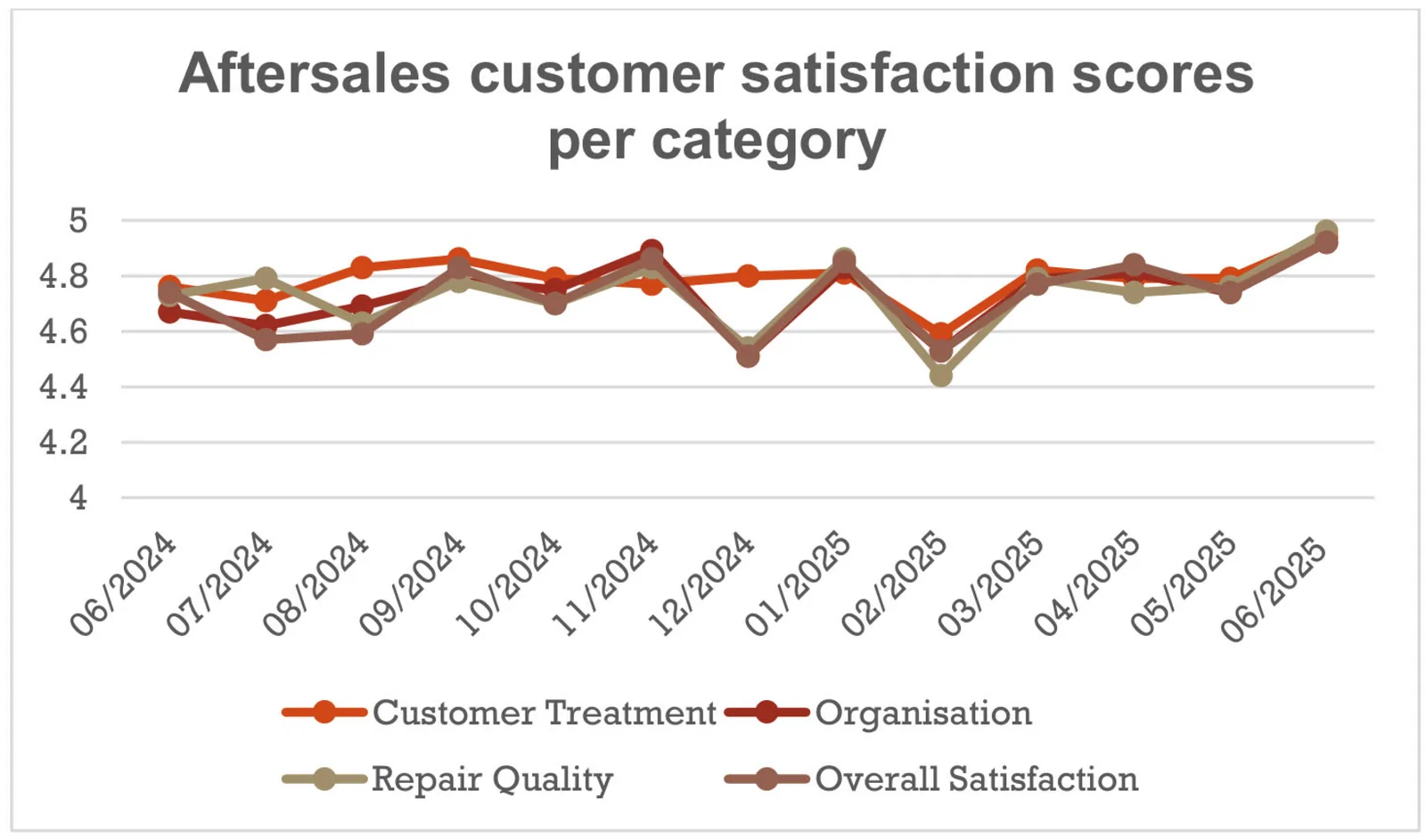
**Customer satisfaction scores at case study** **organisation.**

Aftersales services play a crucial role in the overall business model of automotive dealerships and require continuous improvement [4]; ample opportunities exist to apply creative problem-solving methods to rethink how these services are delivered [5]. A medium-sized (Category 2) dealership in the Western Cape, South Africa, is used as the case study for this research (site description in Section 2.1). During March 2025, the dealership achieved an overall aftersales customer satisfaction score of 4.79 out of 5 against a target of 4.64. Figure 2 presents the dealership’s aftersales customer satisfaction scores across four categories: customer treatment, organisation, repair quality and overall satisfaction, for the period July 2024 to June 2025. Scores remained consistently above 4.5, with customer treatment averaging highest at 4.79 over the period, while organisation, repair quality and overall satisfaction were more variable, each averaging 4.73. The persistent gap between how customers feel they are treated and their satisfaction with repair outcomes suggests that, although service is friendly and professional, the technical and operational aspects of maintenance delivery still require strengthening.

Sustainable waste management is simultaneously becoming more important in the automotive industry [6]. The dealership currently outsources oil waste and other waste streams to a third-party contractor registered under South Africa’s National Environmental Management Waste Act; while compliant, this approach lacks proactive or innovative practices that go beyond basic regulatory requirements. As customers increasingly factor the environmental footprint into purchasing decisions, a more forward-thinking waste management approach is needed to align the business with the values of eco-conscious consumers.

### 1.1. Problem Statement

As customer expectations shift towards efficiency, innovation and sustainability, addressing the identified gaps is essential to improving aftersales service quality. Although the case-study dealership shows strong customer satisfaction overall, gaps remain in maintenance quality and sustainable waste management: customers are treated well but are not always satisfied with repair outcomes, indicating inefficiencies in service delivery, and the current waste management approach, while regulation-compliant, lacks innovation.

### 1.2. Research Opportunity, Aim and Objectives

Biomimicry offers an innovative approach that derives solutions by drawing inspiration from nature [7]. It involves identifying functional challenges in human systems and looking to organisms or ecosystems that have evolved to solve similar problems; because nature’s strategies have been refined over millions of years of evolution, biomimicry often yields solutions that are both efficient and innovative, directly addressing the dealership’s need for improved operational efficiency and proactive waste management.

The aim of this study was to apply biomimicry to increase the operational efficiency of maintenance and repair services and to promote sustainable waste management practices in order to improve overall customer satisfaction at the case-study dealership. Four objectives were pursued: (1) conduct a current state analysis of service activities using functional thinking; (2) apply the biomimicry problem-solving methodology to brainstorm and derive solutions; (3) design a nature-inspired system for aftersales services in the automobile industry; and (4) validate the solution using stakeholder surveys and Life’s Principles.

### 1.3. Scope and Novelty

The study focuses exclusively on the dealership’s automobile aftersales services: vehicle maintenance and repairs, waste management practices, and customer feedback and reporting. Vehicle manufacturing, sales and payment processes fall outside the scope. Within maintenance, the study concentrates on corrective maintenance (repairs performed after a fault occurs) and preventative maintenance (regular servicing to avoid future failures); predictive maintenance falls outside the scope. This project presents the first application of biomimicry within the aftersales services of the selected dealership, and within a service-sector case study in South Africa more broadly. Unlike conventional continuous-improvement approaches, it redesigns service processes using nature-inspired solutions to enhance workflow by addressing efficiency, sustainability and customer satisfaction simultaneously.

## 2. Materials and Methods

### 2.1. Case Study Site

**Figure 3:**
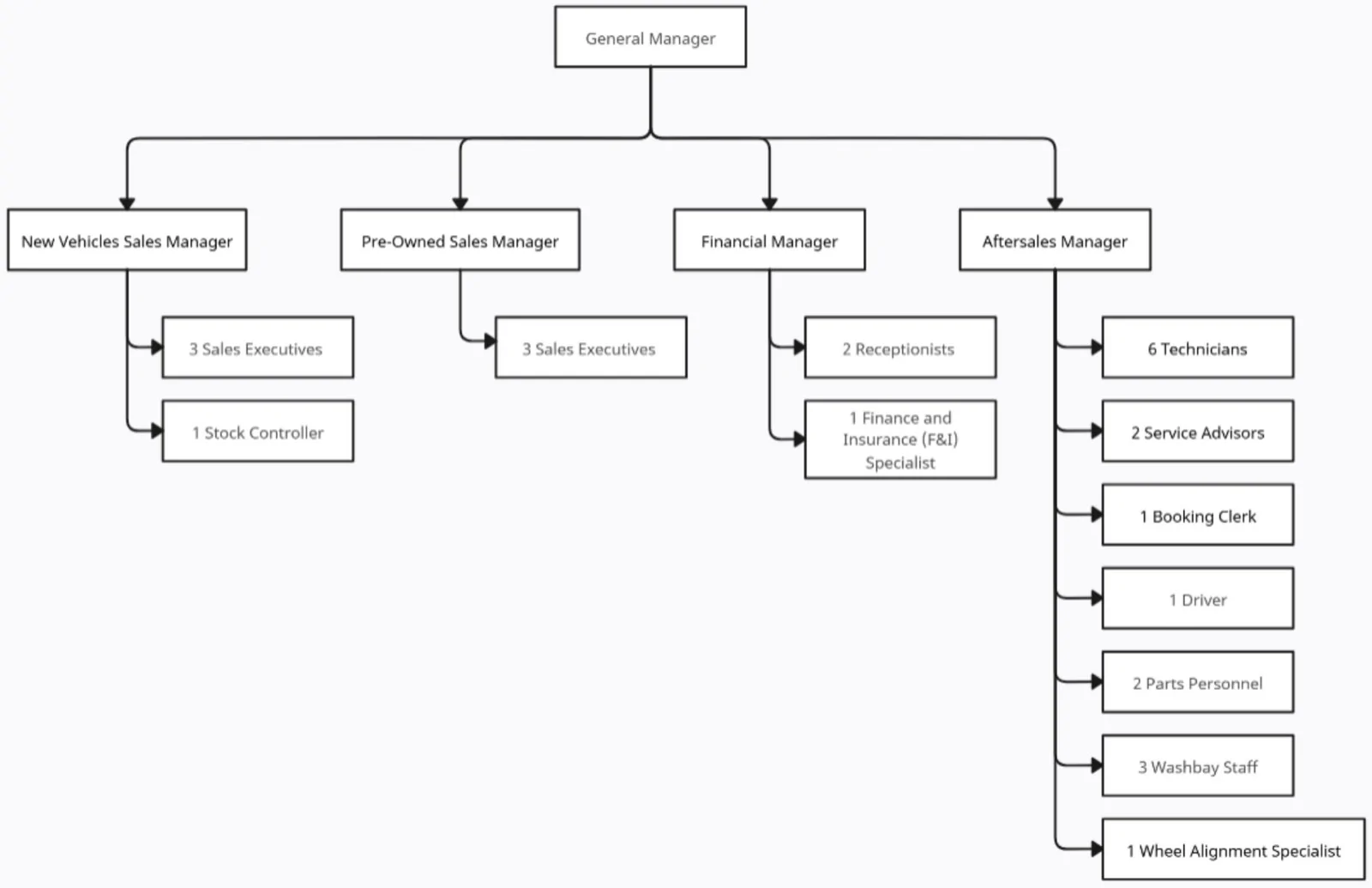
Organogram of the dealership used as the case study.

The case-study dealership is a medium-sized automotive dealership located in the Western Cape, South Africa. The dealership sells between 6 and 15 new vehicles per month and completes between 10 and 20 service jobs per day. The dealership offers new and pre-owned vehicle sales, financing, maintenance plans, genuine parts and motor body repairs, and employs a total of 31 staff. Figure 3 presents the organogram of the dealership generated with draw.io, which is structured into four main areas: New Vehicle Sales, Pre-Owned Sales, Finance and Aftersales.

### 2.2. Theoretical Framework: Biomimicry

Biomimicry, as defined by Benyus [7], is the conscious emulation of life’s genius, or “innovation inspired by nature”. Nature can serve as a model, providing designs and processes that inspire practical solutions; as a measure it sets the standard for what is sustainable and long-lasting after 3.8 billion years of evolution, and as a mentor it encourages a shift from extracting resources to learning from natural strategies. In this study, the term nature-inspired is used interchangeably with biomimicry. The core principle is to emulate nature to solve human problems, since nature has already solved many of the problems that humans currently face, often with a far greater economy of means than human-designed solutions [8]. Bionic (biomimetic) design has been shown to advance sustainable development in industrial design by drawing on the form, structure and function of organisms [9], although translating complex biological strategies into practical solutions remains technically challenging, and market acceptance of biomimicry-based designs can be limited by unfamiliarity [9].

#### 2.2.1. Biomimicry Taxonomy and AskNature

The Biomimicry Taxonomy categorises biological strategies by function to streamline the search for nature-inspired solutions and facilitate biological knowledge transfer [10]. It comprises a three-level hierarchy, Group, Sub-Group and Function, spanning eight groups, 30 sub-groups and more than 160 functions and is searchable through the AskNature platform [11,12]. A recognised limitation is taxonomic bias: biodiversity data over-represent popular species such as birds relative to insects and fungi, which constrains the range of biological strategies considered [13]. Vandevenne et al. [14] demonstrated a scalable approach for automating the classification of biological strategies into the taxonomy, showing how functional challenges such as attachment, movement or protection can be systematically mapped to biological analogies, including in service settings.

AskNature is a curated repository of biological strategies used as inspiration for technological innovation, containing over 200 documented bio-motivated inventions [15]. It was used, for example, in an AI-powered case study assessing how 91 Nature Action 100 companies aligned with the Task Force on Nature-related Financial Disclosures [16], illustrating its value for systematically identifying nature-related strategies and gaps.

#### 2.2.2. Life’s Principles

Life’s Principles are a set of natural design strategies common to every known lifeform, organised around six overarching themes that describe how nature creates “conditions conducive to life” [17]. Integrating these principles into human designs can improve function, resilience and regenerative potential [18]; a case study of social enterprises found that successful ventures consciously or unconsciously employ principles such as optimising rather than maximising, leveraging interdependence and integrating cyclic processes [19]. Applying Life’s Principles can be challenging where traditional practice prioritises standardised, robust methods that conflict with adaptive, nature-based approaches [18]. Nonetheless, the principles provide a proven, evolution-tested framework for validating that proposed solutions are aligned with resilience, adaptability and sustainability [20].

### 2.3. The Automobile Aftersales Service Industry

Aftersales services provide dealerships with a platform to gather ongoing feedback on product and service performance. Adusei and Tweneboah-Koduah [21] found that, in Ghana’s automobile industry, customers who purchase new vehicles are particularly concerned with the aftersales activities provided by the dealer, making aftersales servicing a necessity in a market with a large pre-owned vehicle segment. In South Africa, Mashigo et al. [22] used a qualitative study of dealerships representing several major brands in Gauteng to show that, while literature emphasises quality, reliability and customer experience, some dealerships show a misalignment between strategic objectives and current business models; key challenges included poor service quality, skills shortages, a lack of innovation and gaps in staff training. These studies motivate the use of biomimicry to close such gaps: in maintenance and repairs, a biomimicry-informed design-for-maintenance approach can draw on nature’s “library of knowledge” to create more maintainable, durable and self-sufficient systems [23,24]; in waste management, viewing “waste as a resource”, as demonstrated in a biomimicry-inspired scenic-design case study [25] and in circular-economy research [26], supports more sustainable, revenue-generating waste practices, although scaling such methods can raise logistical and economic challenges [27].

### 2.4. Root Cause Analysis and Solution Development Tools

**Figure 4:**
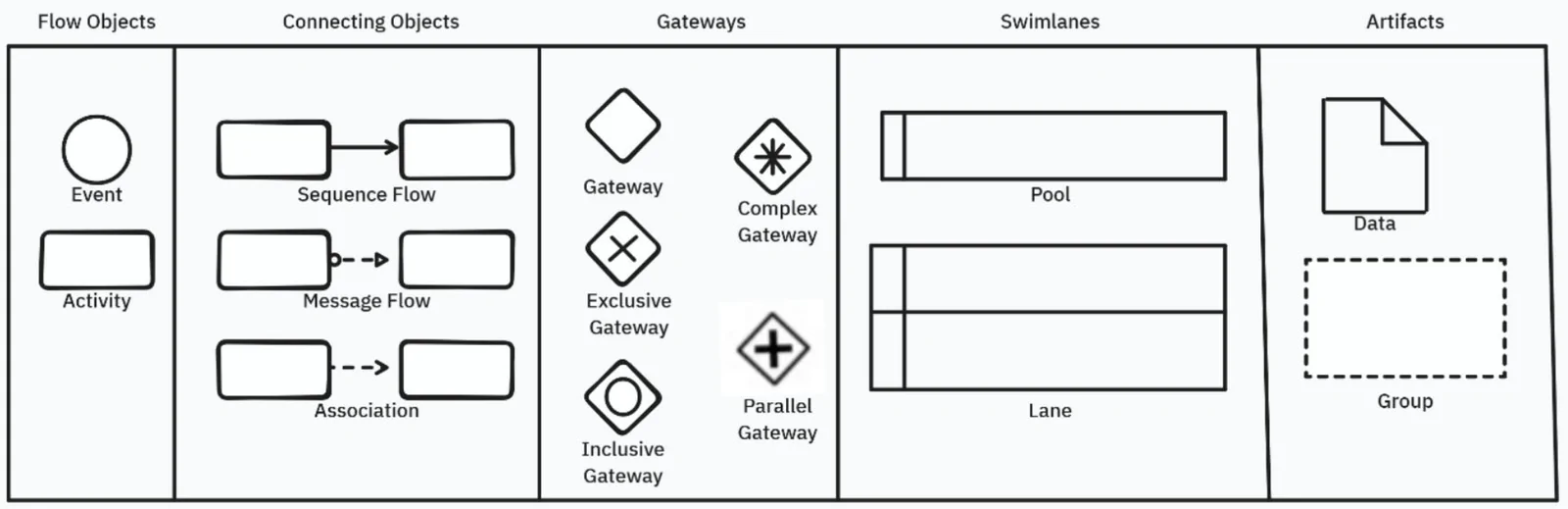
Legend for the Business Process Map notation used in this study.

**Figure 5:**
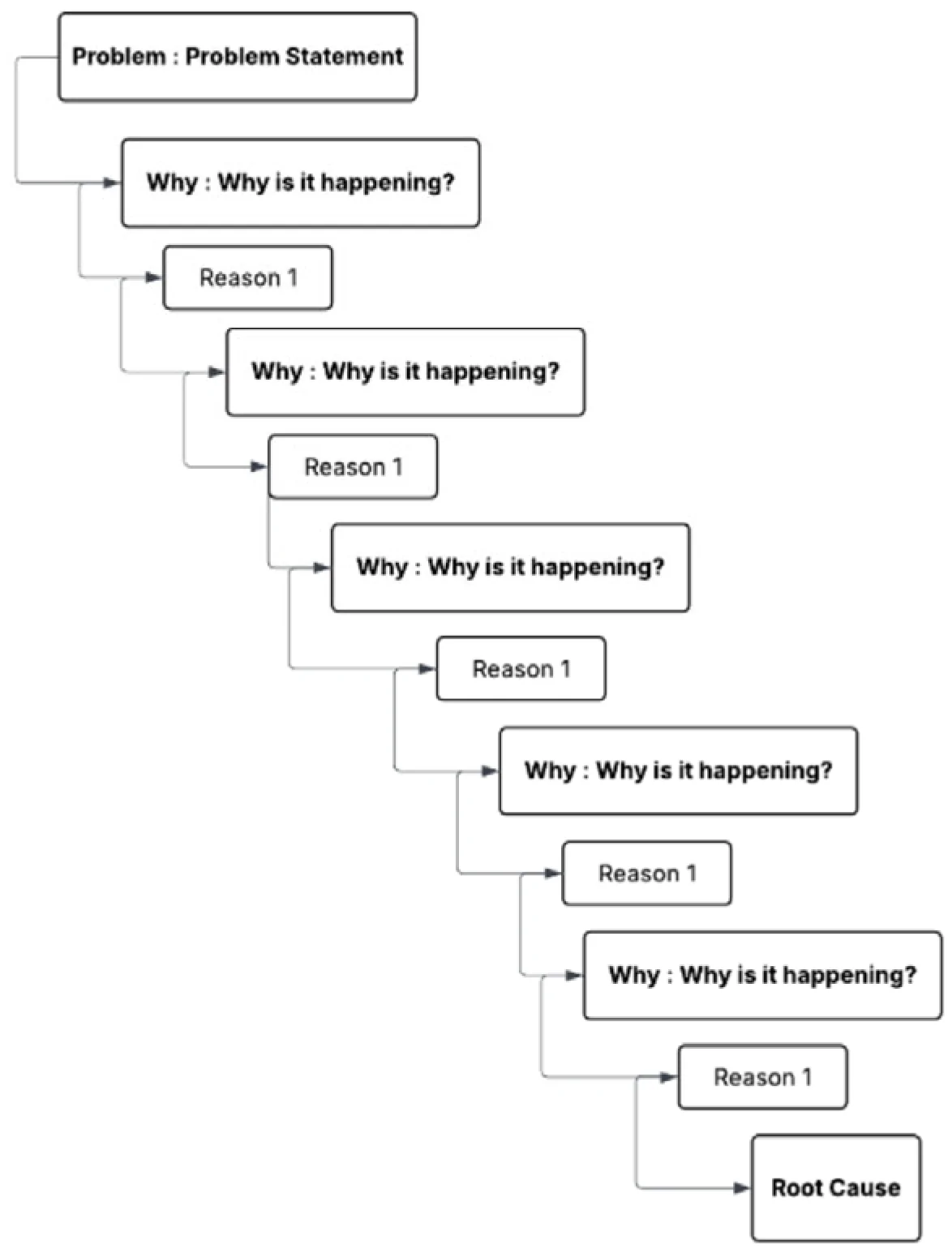
5-Why analysis (Why-Why tree) template.

**Figure 6:**
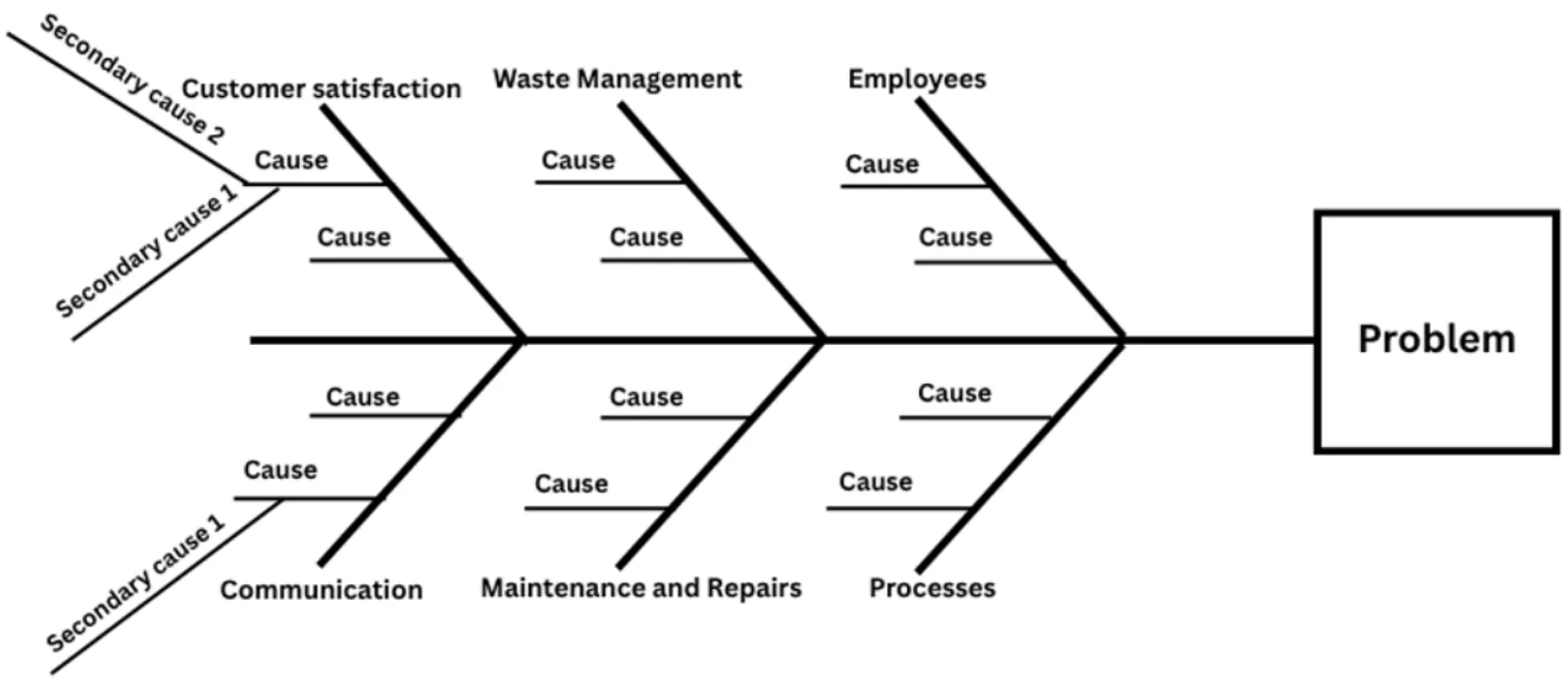
Fishbone (Ishikawa) Diagram template.

A structured root cause analysis identifies the true cause of a problem rather than its symptoms [28]. Three complementary tools were used to analyse the dealership’s current operations. Business Process Mapping (BPM) visualises workflows to expose inefficiencies and organisational barriers [29]; Figure 4 shows the legend used for the process maps in this study, and BPM has previously supported enterprise content management adoption [30] and business process reengineering of automotive aftersales services [31]. The 5-Why technique repeatedly asks “why” to uncover a problem’s root cause [32]; in this study, it was adapted into a Why-Why tree (Figure 5), a branching variant that captures multiple, interrelated causal pathways and is closely related to the Current Reality Tree [33], making it more suitable for the dealership’s multi-causal problems than the traditional linear 5-Why. The Fishbone (Ishikawa) Diagram visualises the relationship between an effect and its contributing causal factors [34]; Figure 6 shows a generic template. Rather than the traditional manufacturing-oriented 6 Ms, categories were customised to reflect the dealership’s service-based operations, since issues in this context relate more to communication, training, system design and culture than to materials or machinery.

**Figure 7:**
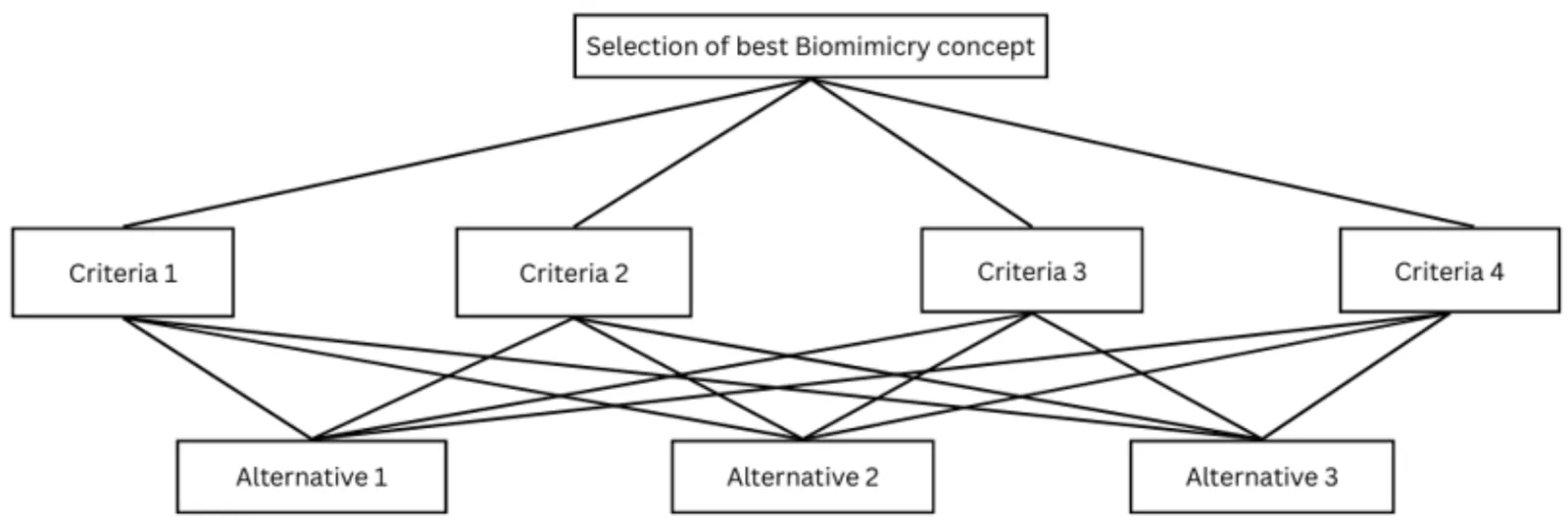
Analytic Hierarchy Process (AHP) template.

Two further tools supported solution development. The Affinity Diagram, devised by Jiro Kawakita [35], uses a bottom-up approach to organise a large number of ideas into hierarchical categories and has been shown to structure complex, subjective information effectively when combined with the Analytic Hierarchy Process (AHP) for prioritisation [36]. The AHP itself is a structured multicriteria decision-making method [37] that decomposes a decision into a goal, criteria and alternatives (Figure 7), uses pairwise comparisons to derive priority weights, and aggregates these into an overall score for each alternative; it has previously been used to build competency models in service industries [38] and to evaluate biologically inspired product designs [39]. Finally, a 5-point Likert scale [40] was used to quantify stakeholder attitudes towards the proposed solution; the scale has demonstrated reliability in translating subjective judgements into data in contexts ranging from clinical imaging [41] to service evaluation.

### 2.5. The Biomimicry Challenge to Biology Methodology

The Biomimicry Challenge to Biology methodology [20] was used as the primary research framework, structuring the study into six stages that move from problem identification to solution verification and validation. Table 1 maps each stage to the corresponding research objective and the tools and techniques applied.

### 2.6. Data Collection and Analysis

Root cause analysis data were derived from direct engagement with the dealership’s operations, process documentation and customer satisfaction records. For validation, identical surveys were distributed via email to the dealership’s Aftersales Manager, technicians and service advisors. Respondents rated their agreement with seven statements, each linked to one of AntLink’s functional requirements, on a 5-point Likert scale, preceded by a general problem-recognition statement and demographic questions capturing age, tenure, role and familiarity with digital workflow tools. Descriptive statistics (means and standard deviations) were computed for each statement, together with a grand mean and mean standard deviation summarising overall response trends.

## 3. Results

### 3.1. Root Cause Analysis

**Figure 8:**
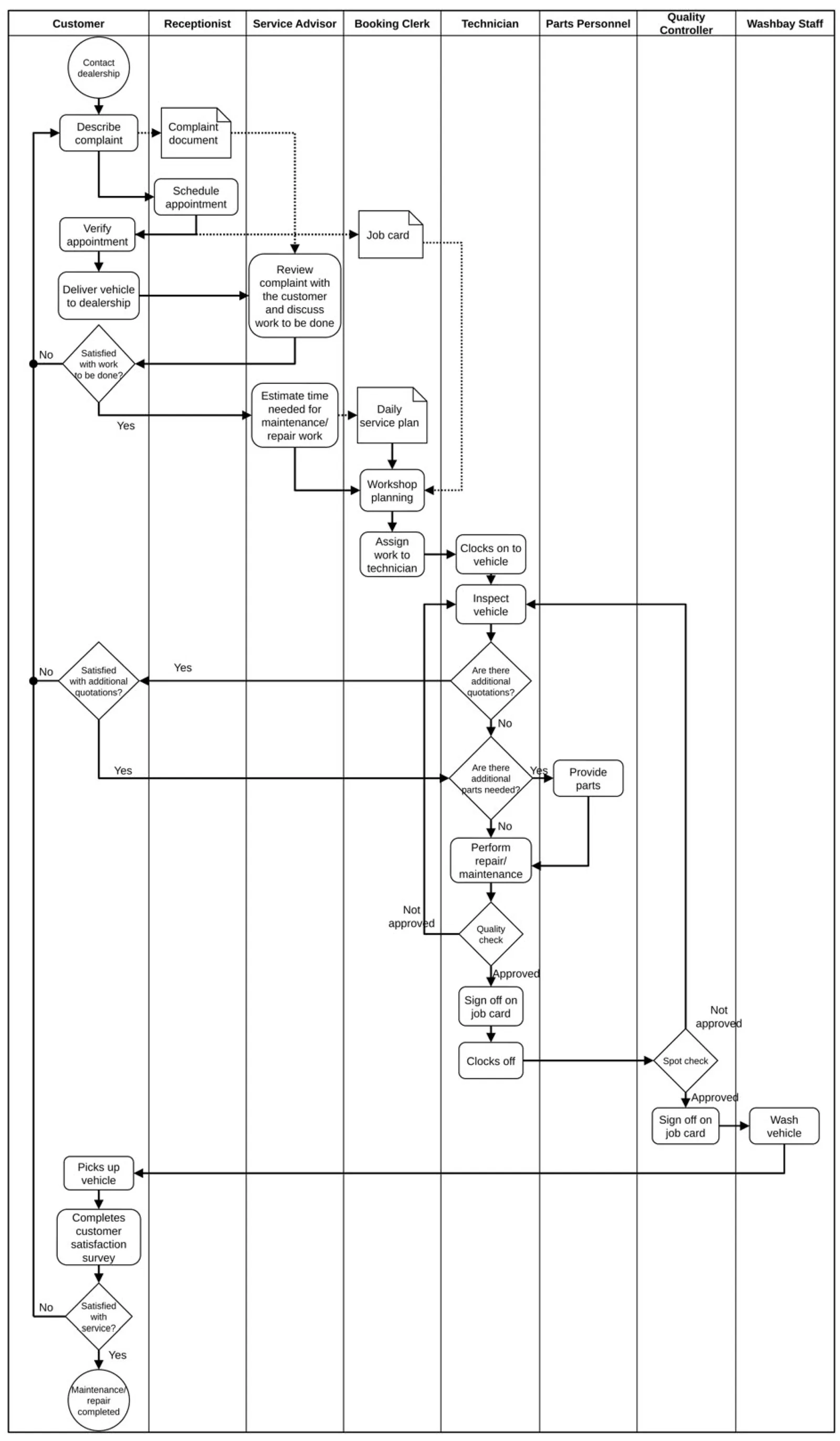
Business Process Map of the dealership’s current aftersales operations.

Figure 8 presents the Business Process Map of the dealership’s current aftersales operations, from customer arrival to service completion, and served as the foundation for identifying inefficiencies in the maintenance and repair workflow. The dealership’s waste management procedure mainly involves the handling of used oil generated from maintenance and repair activities, which is collected and disposed of by a third-party waste management company; while this ensures regulatory compliance, it currently lacks proactive or innovative waste strategies.

**Figure 9:**
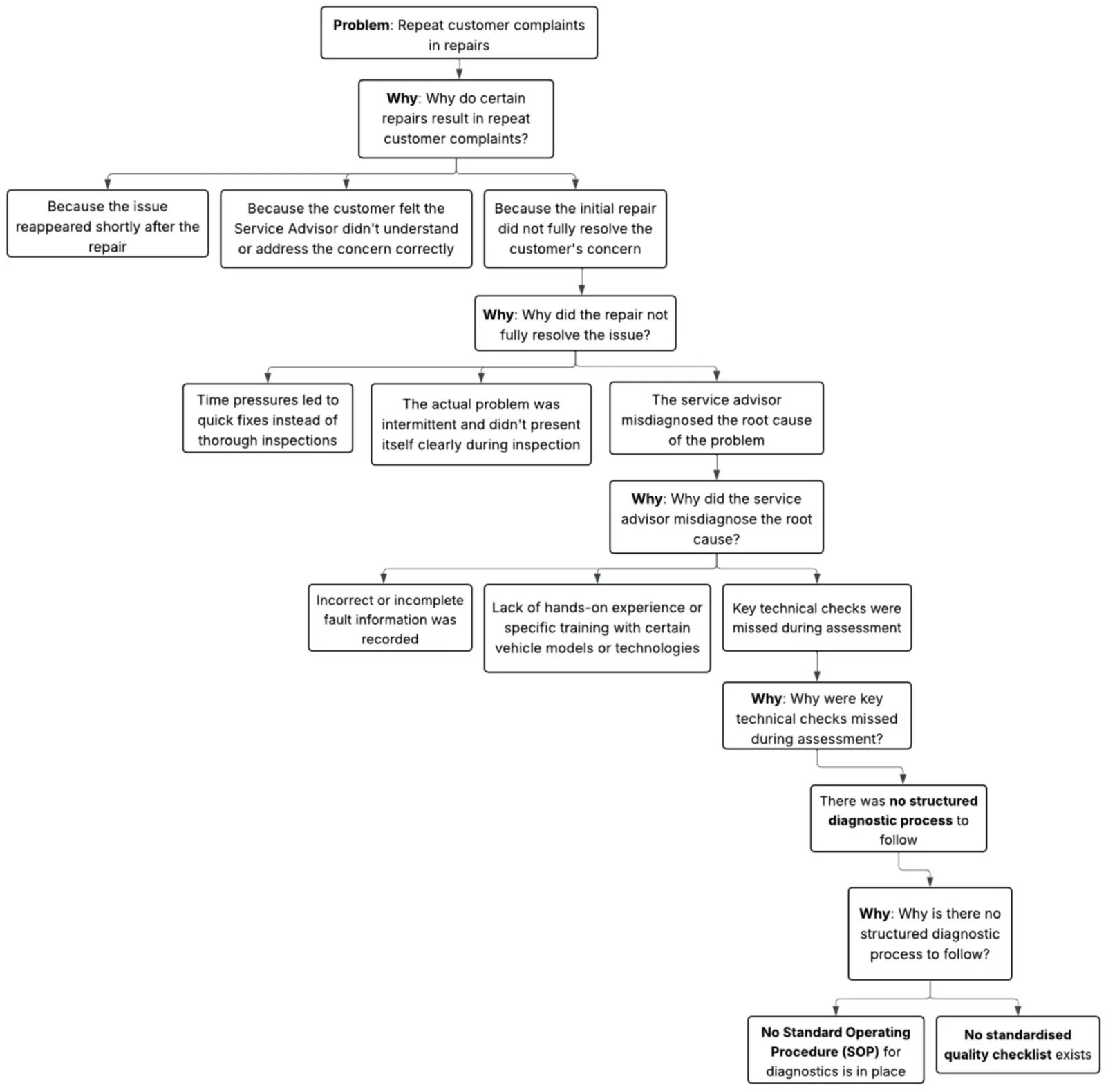
Why-Why tree for maintenance and repair services.

The Why-Why tree analysis was applied separately to maintenance and repairs and to waste management. Figure 9 shows that repeat customer complaints in repairs are driven by a combination of operational, technical and procedural shortcomings: misdiagnosis by service advisors, limited hands-on experience with specific vehicle models, and missed technical checks that stem from the absence of standardised diagnostic procedures and a standard quality checklist. These deficiencies create inconsistencies in fault diagnosis and repair quality, leading to repeat service issues and reduced customer satisfaction.

**Figure 10:**
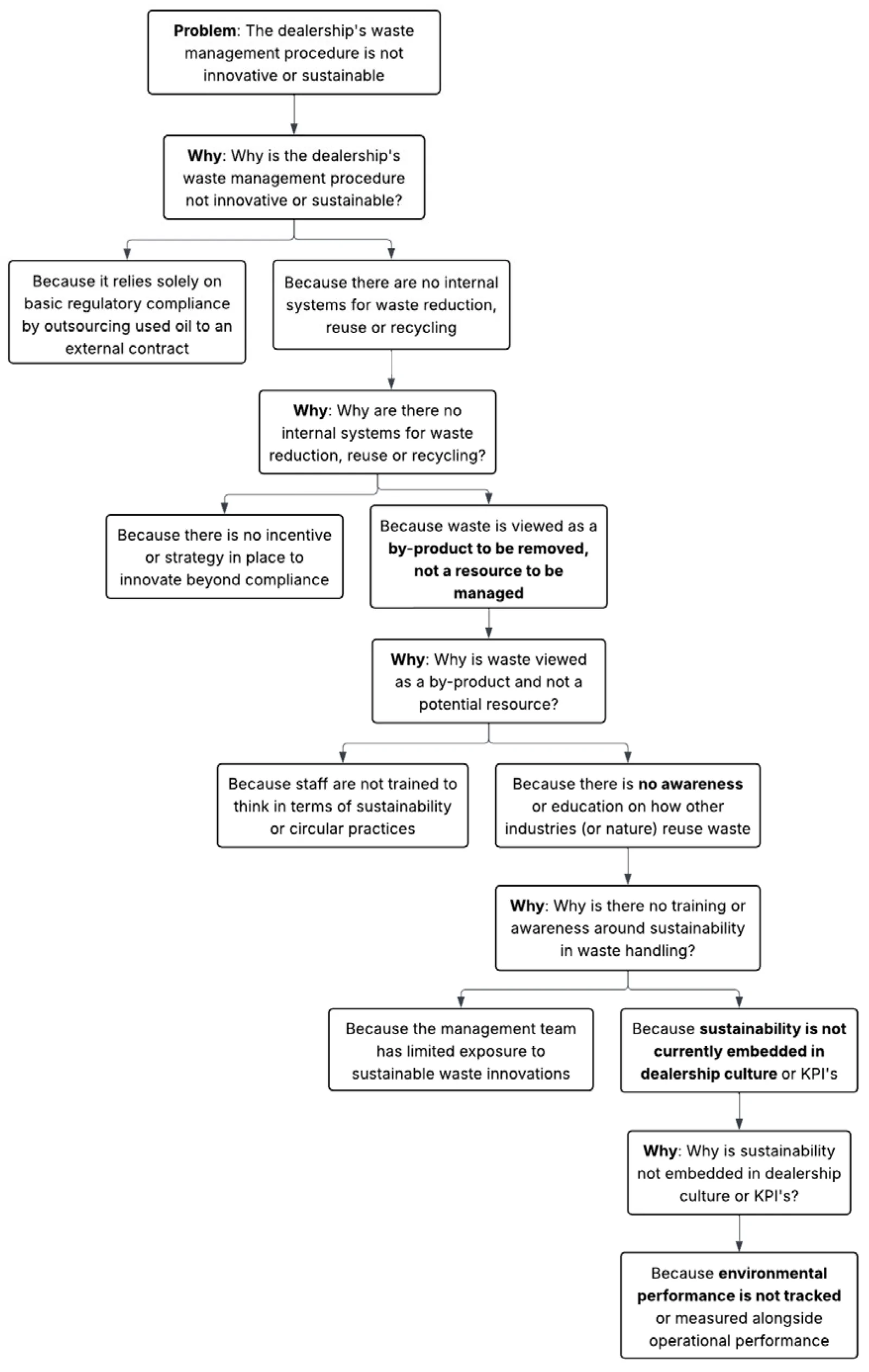
Why-Why tree for waste management procedures.

Figure 10 shows that, in waste management, waste is not viewed as a potential resource at a cultural level, reflecting a linear rather than circular approach. Limited staff training and awareness of sustainable waste principles restrict internal reduction or reuse opportunities, a lack of incentives or performance indicators reduces motivation to improve practices, leadership has not prioritised alternative sustainable solutions, and the current system design lacks internal mechanisms for reuse, leaving the dealership fully reliant on external waste collection.

**Figure 11:**
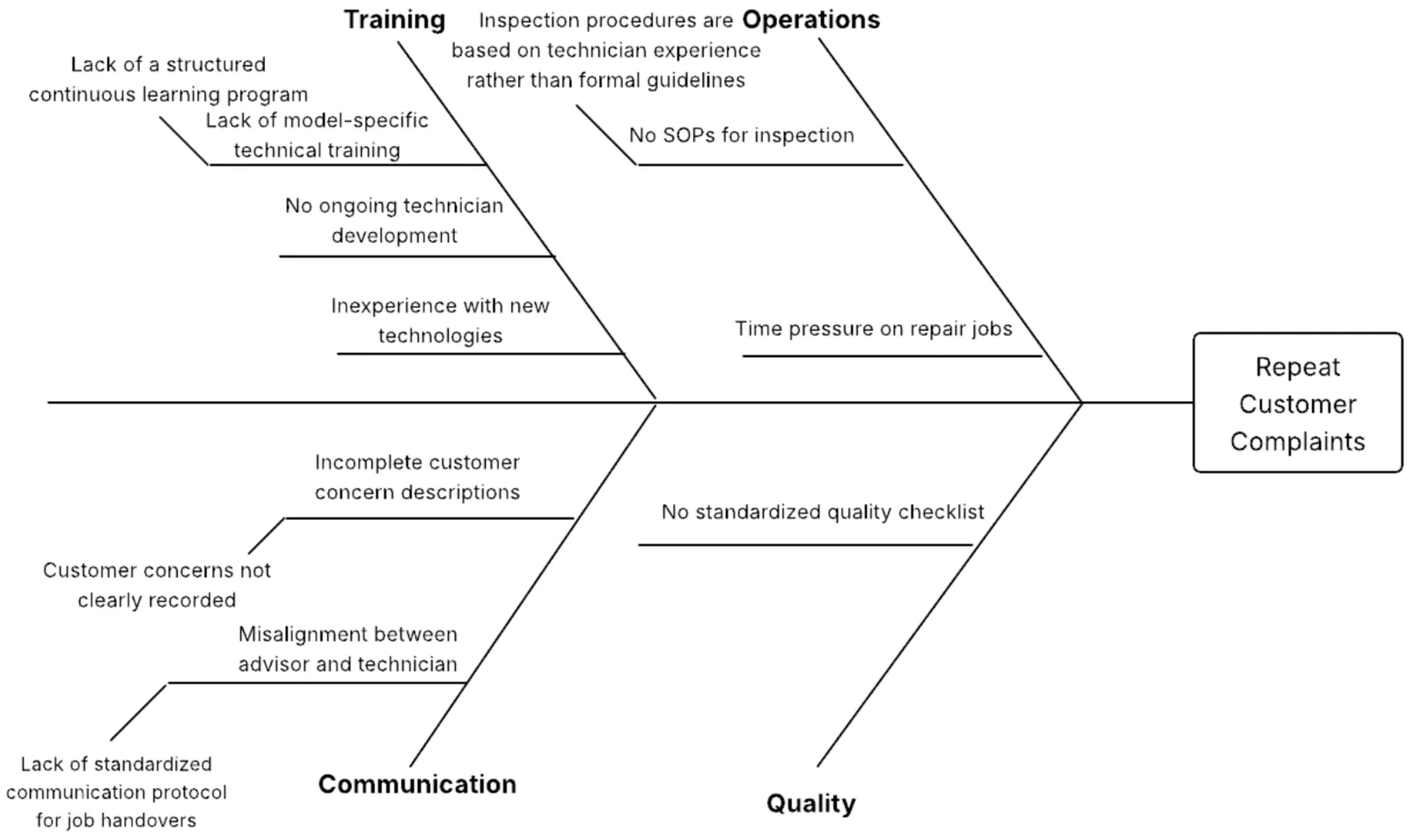
Fishbone Diagram for maintenance and repair inefficiencies.

**Figure 12:**
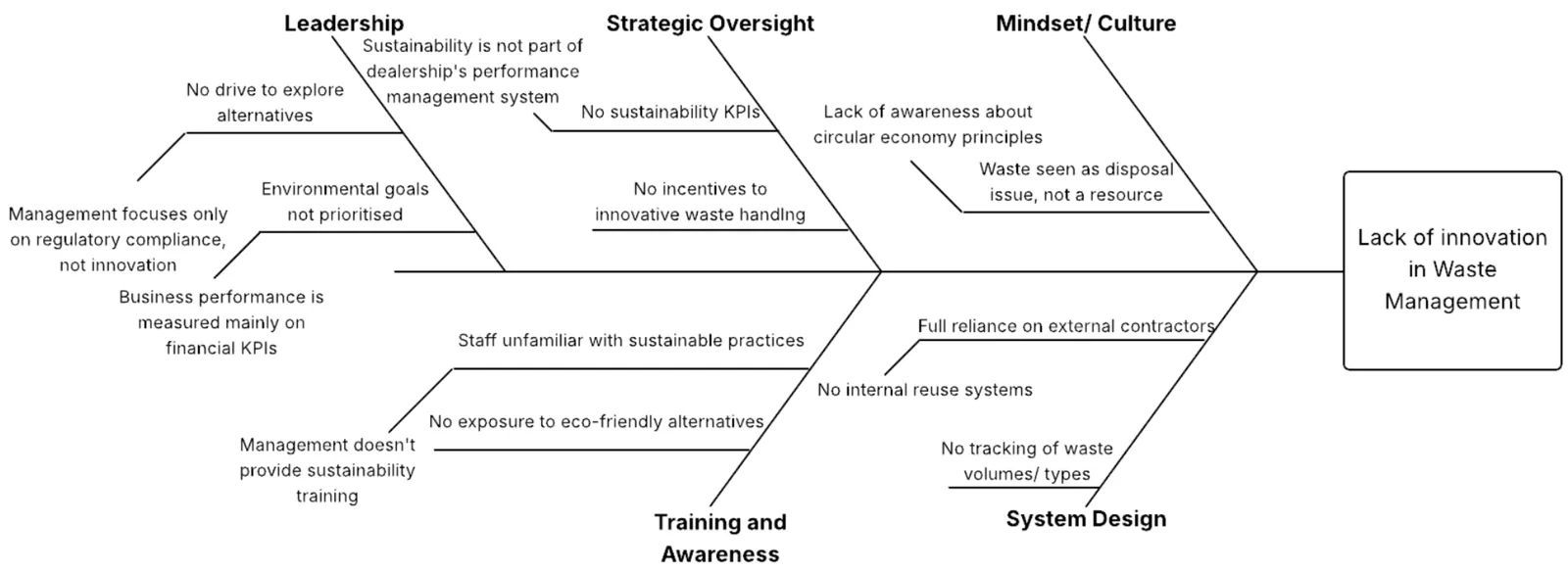
Fishbone Diagram for waste management inefficiencies.

The Fishbone Diagrams in Figure 11 and Figure 12 organise these root causes into clear categories. For maintenance and repairs (Figure 11), poor information flow arises from the absence of feedback loops between technicians and service advisors, inadequate model-specific training stems from a lack of ongoing development programmes, and inconsistent standard operating procedures result from the absence of standardised inspection procedures. For waste management (Figure 12), environmental goals are not prioritised due to a stronger focus on short-term targets, waste is seen purely as a disposal issue due to limited awareness of circular economy principles, and the dealership is fully reliant on external contractors due to the absence of integrated internal platforms.

### 3.2. Concept Requirements and Taxonomy Mapping

**Figure 13:**
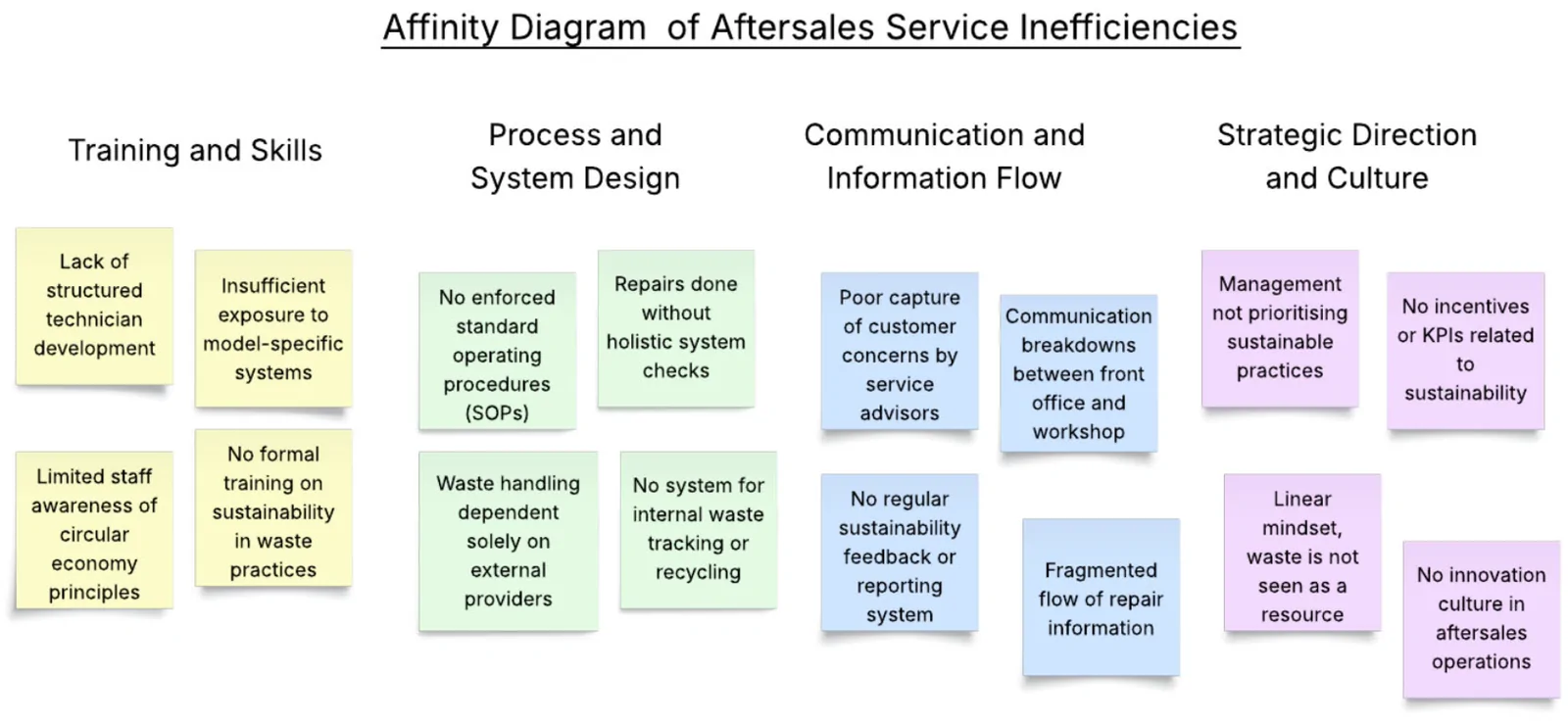
Affinity Diagram of aftersales service inefficiencies.

The root causes identified above were consolidated using an Affinity Diagram (Figure 13), which grouped the dealership’s inefficiencies into four functional areas: training and skills, process and system design, communication and information flow, and strategic direction and culture.

These categories informed a functional design brief (Table 2) that targets improvements in diagnosis and repairs, communication, technician capability, waste reduction and sustainability culture.

Each functional requirement was then mapped to the Biomimicry Taxonomy (Table 3) to derive nature-inspired design principles that guided concept generation.

### 3.3. Three Biomimicry Concepts

Guided by the taxonomy mapping and the AskNature database, three nature-inspired concepts were developed, each targeting specific challenges in maintenance and repair or waste management.

#### 3.3.1. Concept 1: Decentralised Resource Distribution Inspired by Ant Colonies

**Figure 14:**
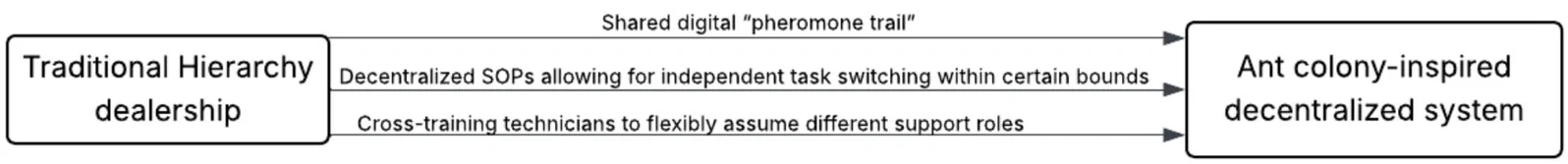
Context-nature transition diagram from a traditional hierarchical dealership model to an ant-inspired decentralised system.

Searching AskNature under “Process Information” and “Process Signals” highlighted ant colonies as an effective model for decentralised communication and resource distribution. Ant colonies coordinate food distribution through local pheromone-based interactions rather than a central control structure, allowing foragers to respond immediately to changing resource availability without the time cost of returning to the nest to recruit others [42]. Translated to aftersales services, this concept encourages service advisors and technicians to collaborate from the start of the customer interaction, supported by standardised forms, so that service concerns are captured consistently and shared across the team. Table 4 summarises the concept’s functional benefits and shortcomings, and Figure 14 illustrates the transition from a traditional hierarchical dealership structure to an ant-inspired decentralised system.

#### 3.3.2. Concept 2: Waste Repurposing Inspired by Fungal Decomposition

**Figure 15:**
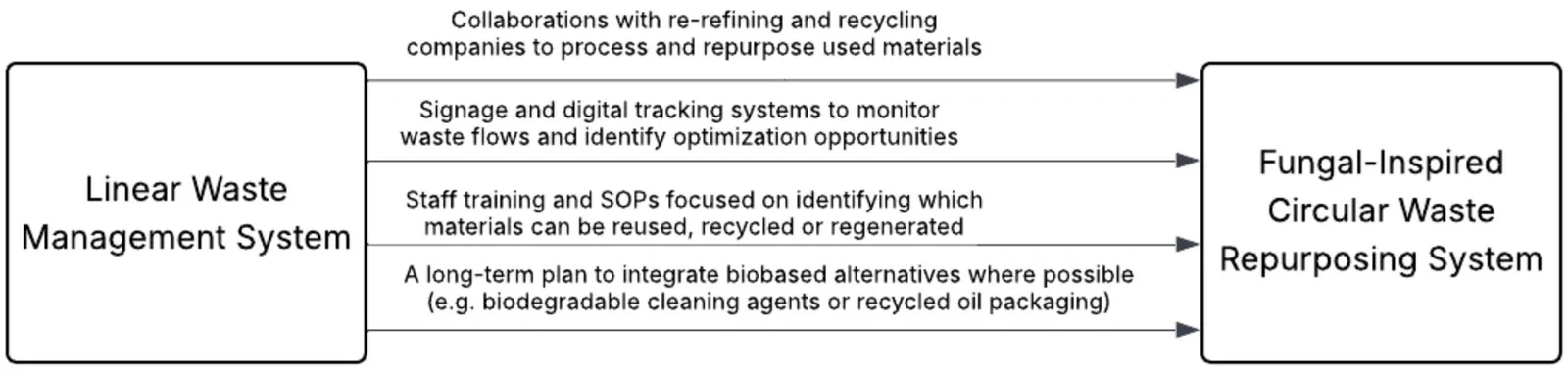
Context-nature transition diagram from linear waste management to circular waste repurposing through a fungal-inspired system.

Searching AskNature under “Modify” and “Adapt/Optimize” highlighted fungi, which decompose complex organic matter and human-made pollutants, including crude oil, into harmless or beneficial substances through mycoremediation, extracting value from waste. Translated to aftersales services, this concept reframes the dealership’s used-oil and waste system through a circular-economy lens: spent oil could be refined into secondary lubricants or industrial fuels through waste-to-energy partnerships, and filters and packaging separated for metal and plastic recovery. Table 5 summarises the concept’s benefits and shortcomings, and Figure 15 illustrates the transition from a linear to a circular, fungal-inspired waste system. However, it should be noted that Concept 2 represents a conceptual framework rather than an implemented operational intervention. True emulation of fungal decomposition mechanisms would require active waste-processing systems (mycoremediation partnerships, re-refining infrastructure) with measurable waste reduction outcomes, not merely tracking and categorisation.

#### 3.3.3. Concept 3: Precision Signal Detection Inspired by Spider Webs

**Figure 16:**
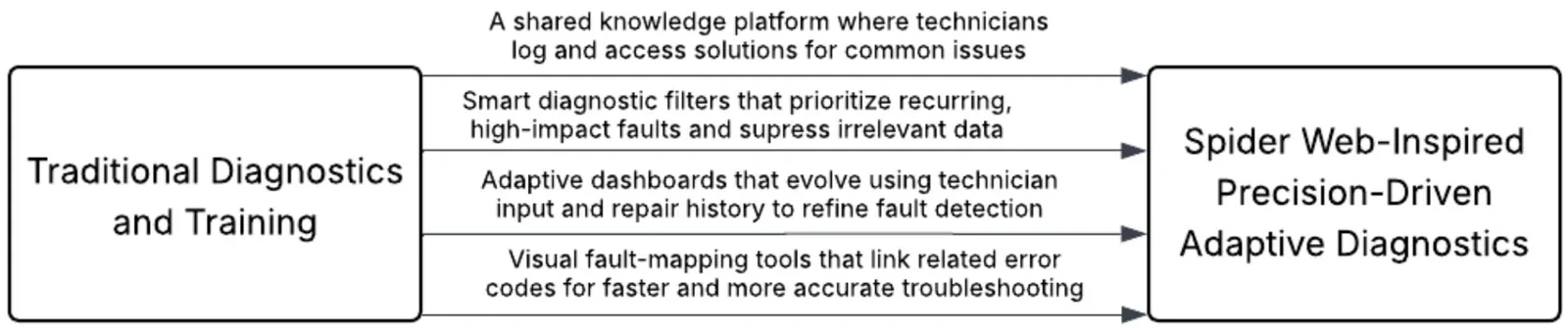
Context-nature transition diagram from traditional diagnostics and training to a spider-web-inspired precision-driven adaptive diagnostics system.

Searching AskNature under “Process Information” and “Process Signals” highlighted spider webs, whose silk and radial structure filter background vibration while amplifying the signal of a struggling insect, enabling precise, rapid responses. Translated to aftersales services, this concept inspires diagnostic tools that emphasise relevant fault signals over low-priority or false alerts, together with adaptive technician-learning systems that track interventions, outcomes and repair times to build feedback loops over time. Table 6 summarises the concept’s benefits and shortcomings, and Figure 16 illustrates the transition from traditional diagnostics and training to a spider-web-inspired, precision-driven adaptive system.

### 3.4. Concept Selection Using the Analytic Hierarchy Process

**Figure 17:**
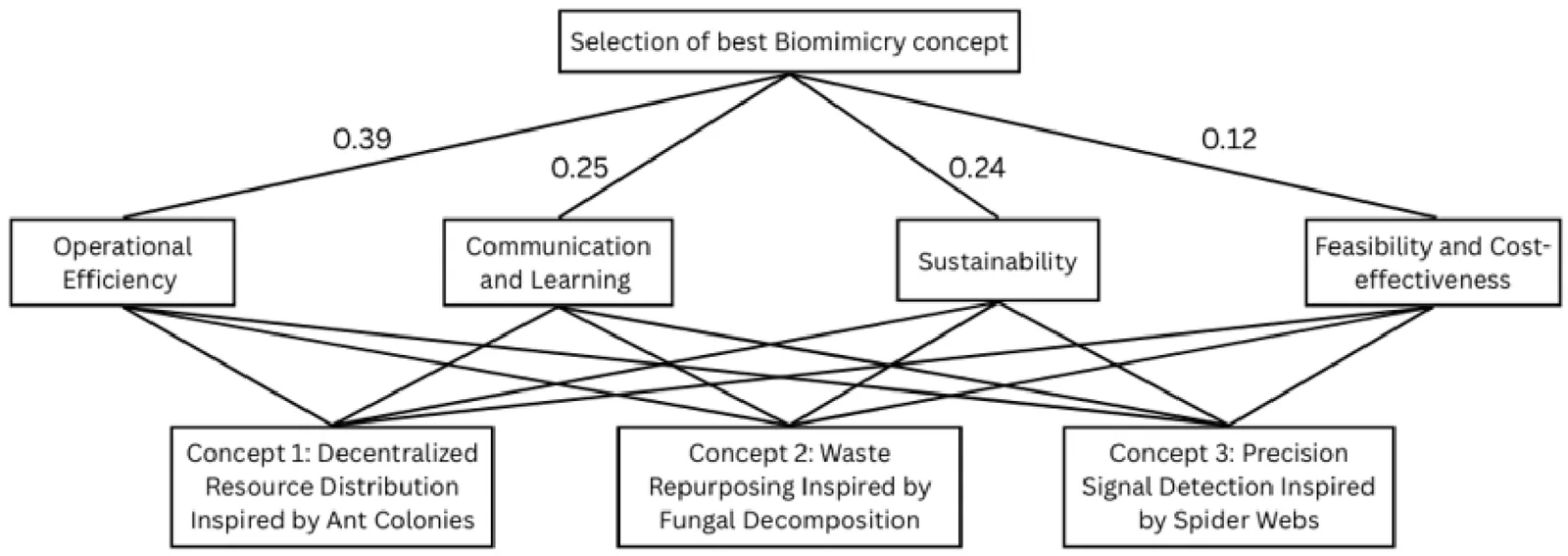
AHP model applied to select the most suitable biomimicry concept for improving aftersales services.

The three concepts were evaluated against four criteria (Figure 17): Operational Efficiency (OE), how well the concept improves speed, reduces downtime and streamlines processes; Communication and Learning (CL), how well it enhances information flow, collaboration and skill development; Sustainability (S), how well it promotes environmental benefit and resource reuse; and Feasibility and Cost-Effectiveness (FC), how practical and affordable implementation would be. Weightings, informed by the root cause analysis in Section 3.1, were configured such that Communication and Learning was equally important as Operational Efficiency, Sustainability was 1.5 times more important than Operational Efficiency, Operational Efficiency was 4 times more important than Feasibility and Cost-Effectiveness, Communication and Learning was 1.1 times more important than Sustainability, and both Communication and Learning and Sustainability were 3 times more important than Feasibility and Cost-Effectiveness.

**Figure 18:**
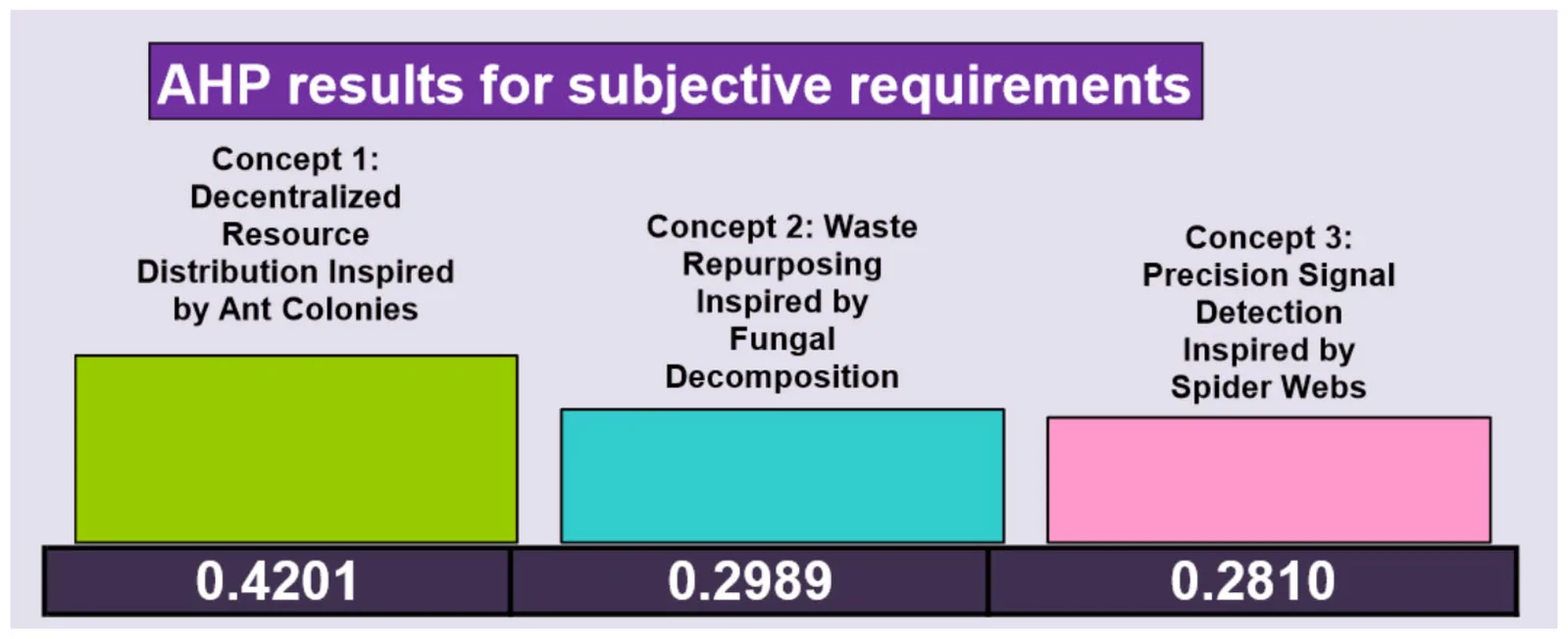
AHP results for the three biomimicry concepts.

Figure 18 presents the AHP results, where Concept 1 (Decentralised Resource Distribution Inspired by Ant Colonies) emerged as the top-ranked choice with an overall score of 0.4201. It excelled in Operational Efficiency, Communication and Learning, and Feasibility and Cost-Effectiveness, directly aligning with the dealership’s primary inefficiencies. Root cause analysis priorities informed the pairwise comparison judgements across four criteria: Operational Efficiency (OE), Communication and Learning (CL), Sustainability (S), and Feasibility and Cost-Effectiveness (FC). The lead researcher evaluated these criteria using Saaty’s nine-point scale. Solving the 4 × 4 pairwise matrix via the eigenvalue method yielded normalised priority weights of S = 0.39, OE = 0.25, CL = 0.25, and FC = 0.11, as reflected in Figure 17. Consistency verification confirmed an acceptable consistency ratio.

### 3.5. Final Design: AntLink

Based on the AHP evaluation, Concept 1 was developed into AntLink, a digital system that introduces a centralised yet collaborative platform where technicians and service advisors can view and contribute to repair data and common faults, reducing process delays and integrating standardised forms for consistent service delivery.

**Figure 19:**
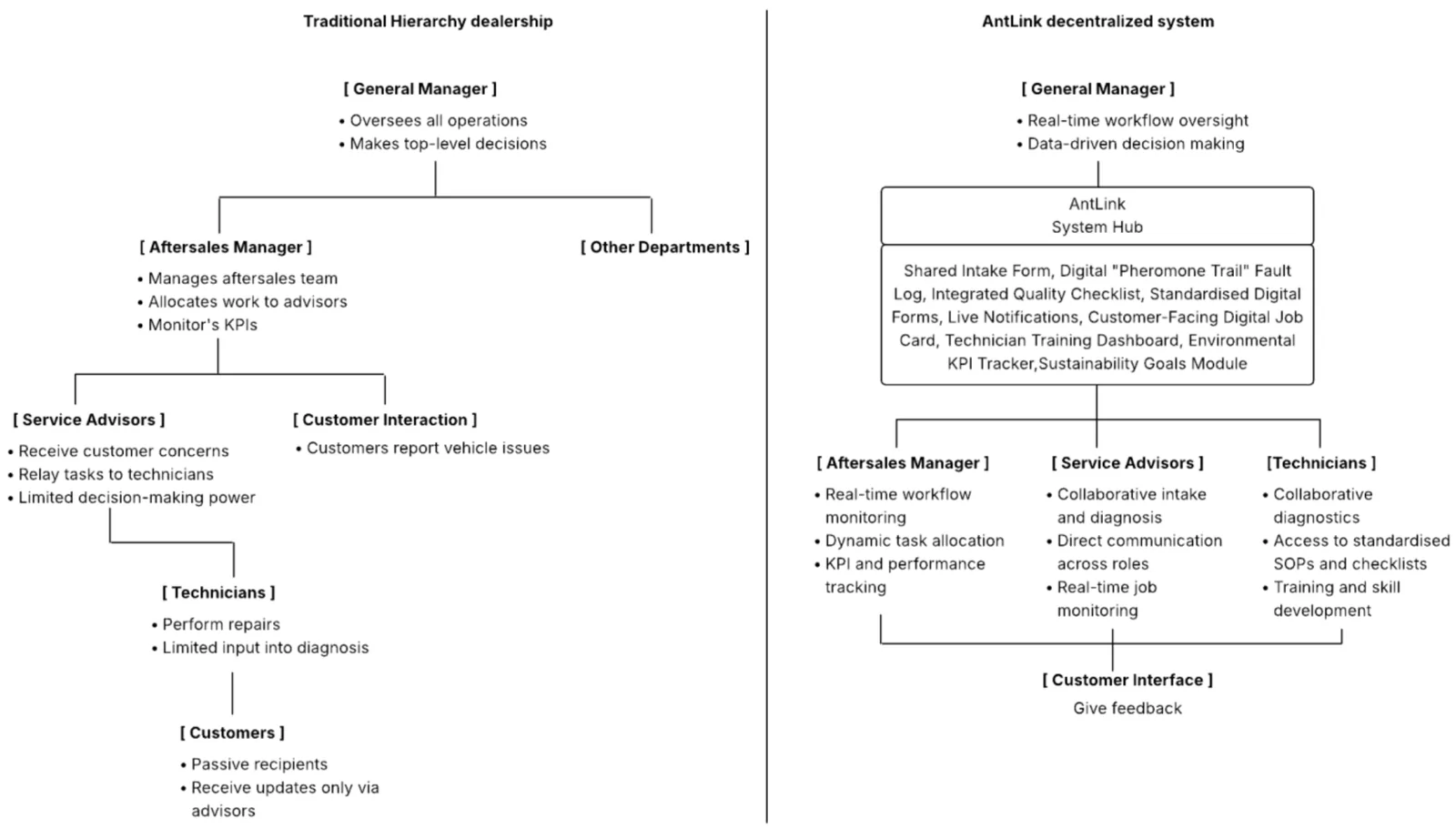
Comparison between the traditional hierarchical dealership structure (**left**) and the AntLink decentralised system inspired by ant colony communication (**right**).

Figure 19 contrasts the dealership’s traditional hierarchy, in which communication is restricted and service advisors often become bottlenecks, with AntLink’s decentralised flow of information, which acts as a digital pheromone trail linking managers, service advisors and technicians in real time and enables dynamic task allocation. Specifically, AntLink decentralises information access and task visibility—technicians and service advisors can directly view and update repair data without requiring approval from the service advisor or manager, mimicking how ant colonies share pheromone information locally for immediate response.

**Figure 20:**
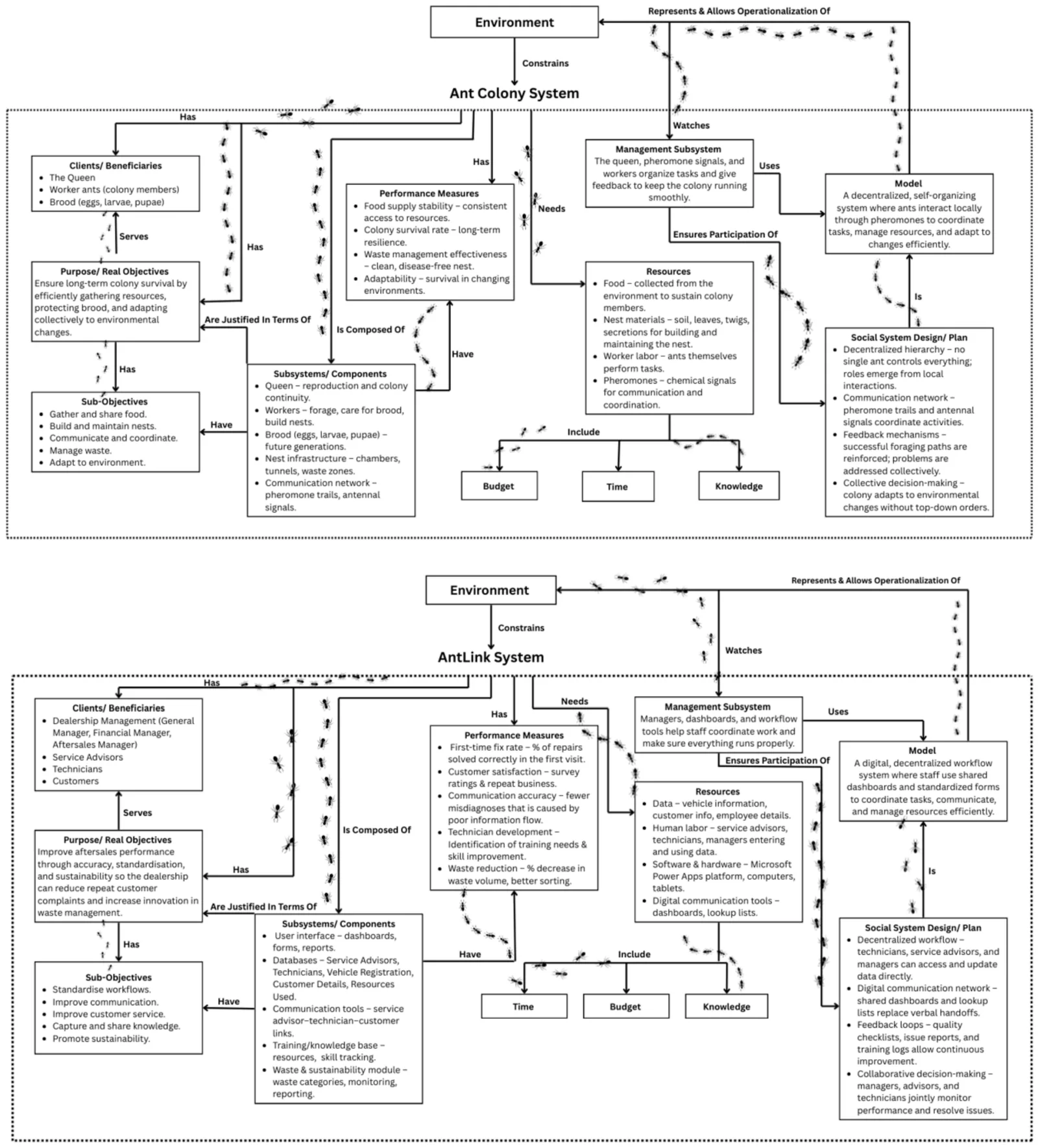
Minimal-system representation, following Churchman [43], of the ant colony system (**top**) and the AntLink system (**bottom**).

Figure 20 presents a minimal-system representation, following Churchman [43], comparing the ant colony system (top) with AntLink (bottom): both rely on decentralised communication rather than rigid hierarchies, with AntLink’s shared dashboards, standardised forms and digital feedback loops playing the role of the pheromone trail that coordinates ant colonies.

**Figure 21:**
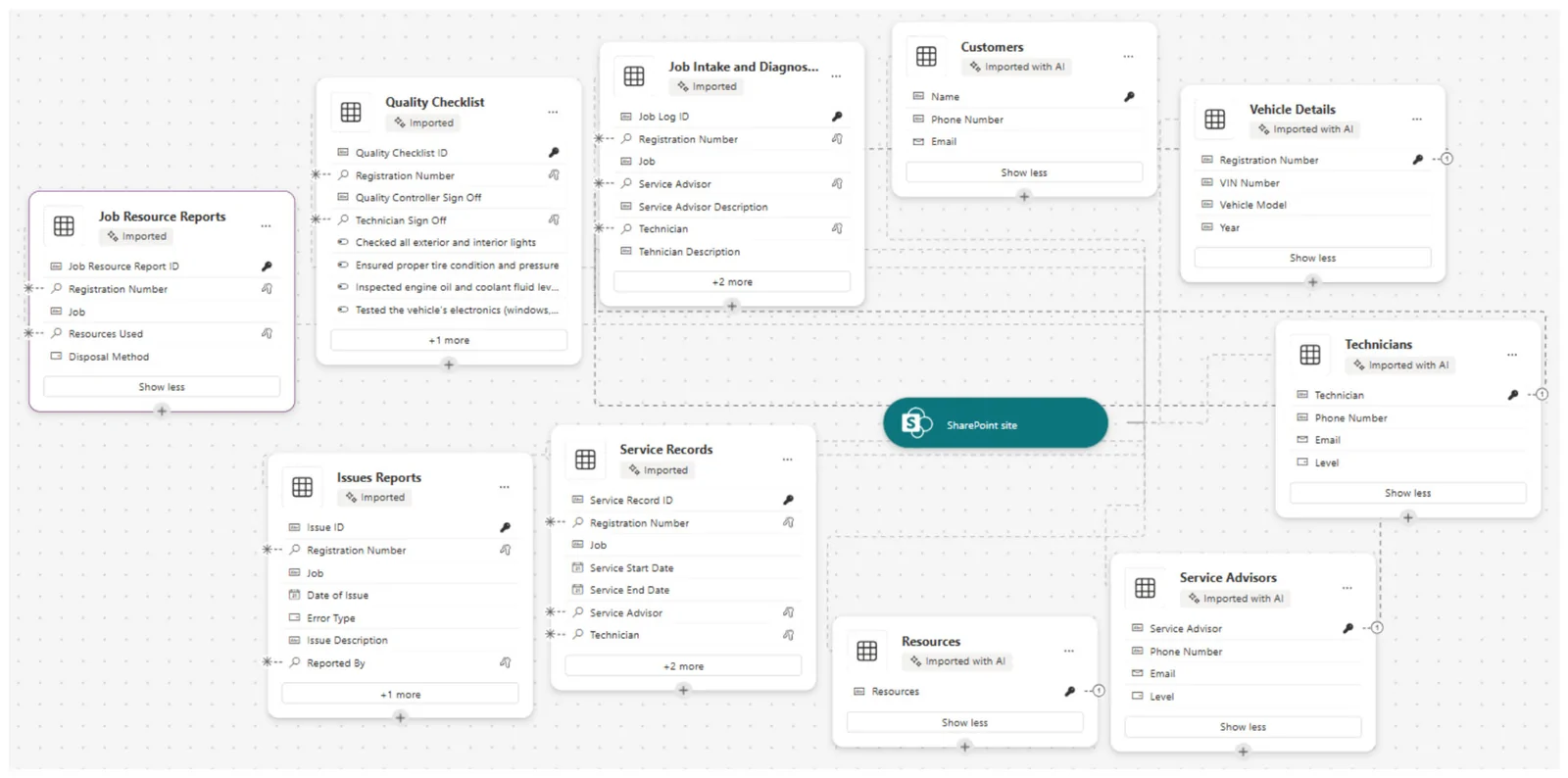
Entity-Relationship Diagram (ERD) for the AntLink digital application.

**Figure 22:**
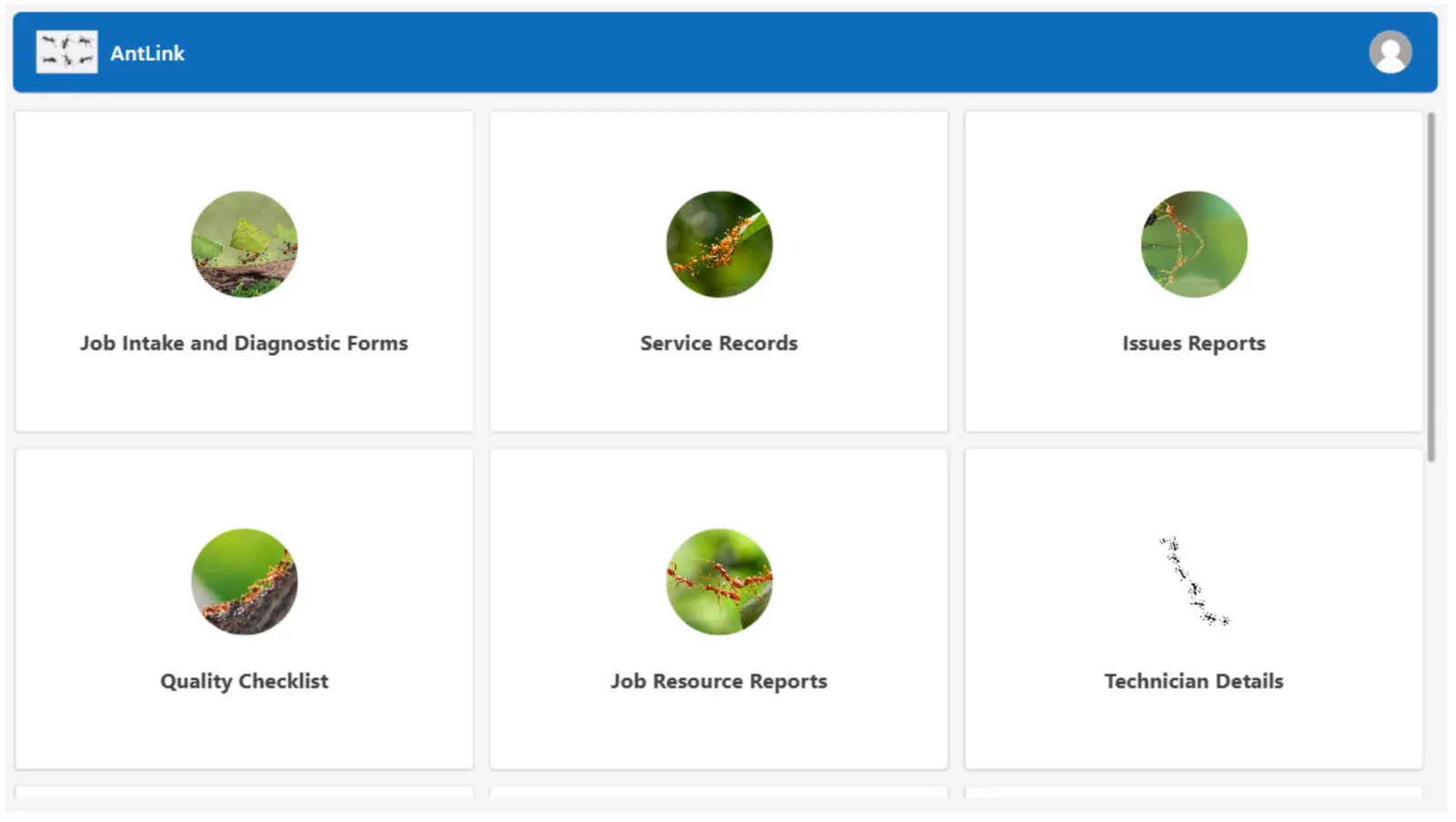
AntLink Home Screen with navigation to dashboards.

The physical design of AntLink began with an Entity-Relationship Diagram (Figure 21) capturing the relationships between service advisors, technicians, vehicles, customers and resources, which formed the data architecture for five main operational dashboards (intake and diagnostics, issue reports, service tracking, quality control and resource management) and five supporting dashboards for reference data. The application was developed iteratively in Microsoft Power Apps, with each dashboard interacting dynamically with related data tables to maintain consistency across the system. Figure 22 shows AntLink’s Home Screen, which provides navigation to the main and supporting dashboards.

### 3.6. Verification and Validation

#### 3.6.1. Verification Against Functional Requirements

Table 7 maps each functional requirement to its AntLink feature implementation and the corresponding ant-colony biomimicry principle. Verification confirmed that AntLink’s features effectively satisfy all seven functional requirements, embedding both operational improvements and ecological awareness into the dealership’s aftersales operations.

#### 3.6.2. Stakeholder Survey Validation

Identical surveys were distributed via email to the dealership’s Aftersales Manager, service advisors and technicians, and eventually a sample of nine participants. These participants rated seven statements, each linked to an AntLink functional requirement, on a five-point Likert scale, following an initial problem-recognition statement. Table 8 reports the descriptive statistics.

The grand mean of 3.958 indicates an overall positive level of agreement that AntLink meets its intended functional requirements, and the mean standard deviation of 1.000 suggests relatively consistent perceptions across participants. The problem statement received a mean of 3.833 with a comparatively high standard deviation (SD = 1.169), suggesting that while the problem addressed by AntLink is broadly recognised, a small portion of respondents may need further engagement to align with the problem context. The seven validation statements showed consistent agreement, with means a range from 3.500 to 4.333; the highest level of agreement (M = 4.333, SD = 0.516) concerned the built-in quality checklist, indicating strong confidence in this core function, while communication efficiency and skill development statements both scored M = 4.167 with low variability. The lowest mean score (M = 3.500) concerned the shared intake and diagnostic forms; its elevated standard deviation (SD = 1.517) was largely attributable to a single outlying respondent rating it 1, suggesting overall agreement with this feature remains acceptable. Respondents had a mean age of 40.833 years (range 29–50), an average of 8.958 years of service at the dealership (SD = 11.325) and 19.500 years of experience in the automotive aftersales industry on average (SD = 9.482); 50% reported high familiarity with digital workflow tools, 33.33% low familiarity and 16.67% moderate familiarity, suggesting that some staff may require support during implementation.

#### 3.6.3. Validation Against Life’s Principles

AntLink’s conceptual design was evaluated against Biomimicry 3.8’s Life’s Principles [17] to assess its potential to be sustainable, resilient and adaptive. Table 9 summarises how the system reflects each principle and its relevant sub-principles.

Validating AntLink against Life’s Principles confirmed that its design incorporates nature-inspired strategies such as modularity, feedback, resilience and efficiency, suggesting the solution is not only operationally viable but also conceptually sustainable, supporting its long-term integration into the dealership’s aftersales processes. This complementary validation approach strengthens the study by providing both stakeholder-centred evidence of usability and acceptability through survey metrics, and design-centred evidence of alignment with natural strategies through Life’s Principles assessment. Together, these methods establish that AntLink is not only perceived as useful by the operational team but also conceptually grounded in nature-inspired sustainability principles.

## 4. Discussion

The results demonstrate that the Biomimicry Challenge to Biology methodology can be applied systematically to a real-world automotive aftersales operation, translating soft, exploratory root cause findings into a verifiable, quantitatively validated digital solution. The selection of ant colony organisation as the leading biomimicry concept is consistent with prior findings that decentralised, pheromone-mediated coordination in ant colonies enhances a system’s speed, resilience and adaptability under changing conditions [42]; AntLink’s positive verification and validation results (grand mean 3.958) suggest that these properties translate meaningfully into a service-operations context. This complements the existing aftersales literature, which has identified poor service quality, skills shortages and a lack of innovation as recurring challenges in South African dealerships [22] and has called for innovation incentives, staff training and technology adoption as remedies; AntLink operationalises several of these recommendations directly through shared diagnostic forms, quality checklists and training-needs dashboards.

The strongest stakeholder agreement was recorded for AntLink’s built-in quality checklist (M = 4.333), while the shared intake and diagnostic forms scored comparatively lower and more variably (M = 3.500, SD = 1.517), indicating that although a structured checklist is readily accepted, changes to how service advisors and technicians jointly capture customer concerns may require more deliberate change management. This nuance echoes Bergsma’s [23] observation that biomimicry-based frameworks in maintenance contexts are useful for organising the design process but benefit from stronger emphasis on the feedback phases of implementation. The waste-management concept selected through the AHP (ant-inspired decentralised resource distribution) was prioritised over the fungal-inspired waste repurposing concept because the weighting criteria emphasised operational efficiency and communication over sustainability alone; this is not to suggest that circular-economy opportunities identified in Concept 2 are without merit; rather, AntLink’s job resource reporting dashboard incorporates a subset of this concept’s waste-tracking logic, consistent with the literature showing that viewing “waste as a resource” supports both sustainability and additional revenue streams [25,26]. While full implementation testing is beyond the scope of this prototype evaluation, these comparable studies support the potential for AntLink to deliver measurable operational improvements upon deployment. It is important to acknowledge that while AntLink includes waste tracking and categorisation capabilities through the job resource report dashboard, the selected Concept 1 (decentralised resource distribution) does not directly implement waste reduction, reuse, or re-refining interventions. Tracking and monitoring are enablers that support future waste improvement initiatives, but they do not themselves constitute evidence of sustainable waste management. A complete waste-management solution would require operational interventions with specific baselines, targets, assigned responsibilities, and measured outcomes—such as partnerships with waste-to-energy providers, established recycling protocols with monthly volume reduction targets, or staff incentive programmes tied to waste reduction metrics. Concept 2’s fungal-inspired approach offers this level of operational intervention but was not selected in this iteration due to implementation feasibility constraints. Future work should investigate whether combining Concept 1’s decentralised communication with Concept 2’s circular-economy waste interventions would strengthen both operational efficiency and sustainability outcomes.

Several limitations should be noted. Verification and validation relied on stakeholder surveys and the Life’s Principles framework rather than full implementation; longitudinal tracking of key performance indicators such as customer callback rates and waste reduction volumes was outside the study’s scope and is necessary to quantify AntLink’s return on investment. The survey sample, while representative of the dealership’s aftersales roles, was small, and the elevated standard deviation on the intake-and-diagnostic-forms statement was substantially influenced by a single outlying response. The study’s boundaries also excluded vehicle manufacturing, sales and payment processes, meaning AntLink’s value proposition, while strong within aftersales, has not been assessed for integration with upstream dealership functions such as procurement or financing.

## 5. Conclusions and Recommendations

This study applies the Challenge to Biology methodology to optimise maintenance, repair, and waste operations in a medium-sized automotive dealership. Root causes of operational and sustainability challenges were identified through functional-thinking tools and mapped to the Biomimicry Taxonomy. Using the Analytic Hierarchy Process, ant colony-inspired decentralised distribution was selected to create AntLink—a digital system establishing a real-time “pheromone trail” across managers, advisors, and technicians. AntLink replaces rigid hierarchies with collaborative intake, diagnostic, quality, and waste-tracking tools, directly resolving misdiagnoses, inconsistent procedures, and sustainability gaps. The system satisfied all functional requirements, achieving strong operational feasibility (3.958/5 stakeholder score) and conceptual sustainability via Life’s Principles, with future work targeted at longitudinal tracking of callbacks and waste reduction.

## Figures and Tables

**Table 1:** Biomimicry Challenge to Biology stages with corresponding objectives and tools and techniques used.

Biomimicry Challenge to Biology Stage	Objective	Tools and Techniques
Identify—Real Challenge	Objective 1: Conduct a current state analysis on service activities using functional thinking.	Literature review; Business Process Map; 5-Why Analysis (Why-Why tree); Fishbone Diagram
Interpret—Design Brief	Objective 2: Apply the biomimicry problem-solving methodology to brainstorm and derive solutions.	Affinity Diagram
Abstract—Design Principles		Biomimicry Taxonomy
Discover—Natural Models		AskNature; three biomimicry concepts; AHP design selection method
Emulate—Nature’s Strategies	Objective 3: Design a nature-inspired system for aftersales services.	Emulation of the selected concept into a nature-inspired framework
Evaluate—Against Life’s Principles and Stakeholder Surveys	Objective 4: Validate the solution using surveys and Life’s Principles.	Surveys (5-point Likert scale); Life’s Principles

**Table 2:** Functional design brief with requirements and specifications for aftersales service improvement.

Category	Functional Requirement (FR)	Design Specification
Diagnosis and Repairs	FR1: Accurate fault diagnosis.	≥95% of repairs should resolve issues on the first visit.
	FR2: Consistent repair quality.	Implement a digital diagnostic checklist/system within 3 months.
	FR3: Increase process standardisation.	Standardised forms created for ≥85% of recurring service processes.
Communication	FR4: Effective communication of service issues.	Use standardised intake forms and concern-capture templates.
Technician Capability	FR5: Technician capability development.	≥1 structured technician training session per technician per quarter.
Waste Reduction	FR6: Reduce waste volume and improve sorting.	Implement ≥ 3 waste categories with monthly monitoring.
Sustainability Culture	FR7: Encourage a sustainability mindset.	Conduct ≥ 2 sustainability awareness workshops per year.

**Table 3:** Functional requirements mapped to the Biomimicry Taxonomy and translated into nature-inspired design principles.

Functional Requirement	Biomimicry Taxonomy Mapping	Nature-Inspired Design Principle
FR1: Accurate fault diagnosis.	Process information → process signals → respond to signals	Design systems that filter background noise and highlight critical signals to improve diagnostic accuracy.
FR2: Consistent repair quality.	Protect from physical harm → manage structural forces	Emulate natural structures that manage stress and maintain reliability under pressure.
FR3: Increase process standardisation.	Make → re-produce → self-replicate	Model processes after biological systems that replicate with precision and consistency.
FR4: Effective communication of service issues.	Process information → Process signals → Transduce/convert signals	Mimic organisms that convert complex inputs into clear, simple messages that can be acted upon.
FR5: Technician capability development.	Process information → learn	Create systems that support continuous learning and adaptation through feedback.
FR6: Reduce waste volume and improve sorting.	Modify → adapt/optimise → adapt behaviours	Emulate organisms that adapt behaviours to optimise resource use and reduce waste.
FR7: Encourage a sustainability mindset.	Modify → adapt/optimise → optimise space/materials	Design for minimal, efficient use of materials and space—doing more with less.

**Table 4:** Functional benefits and potential shortcomings of Concept 1 (ant-inspired decentralised resource distribution).

Functional Benefits	Potential Shortcomings
Faster and more accurate diagnoses and repair response.	Without clear hierarchy, some tasks may be duplicated or overlooked.
Improved internal communication and information flow.	Autonomy can lead to varying interpretations of procedures if not standardised effectively.
Minimised downtime due to bottlenecks in decision approval.	Cross-training staff to be flexible in multiple roles requires time, resources and ongoing commitment.

**Table 5:** Functional benefits and potential shortcomings of Concept 2 (fungal-inspired waste repurposing).

Functional Benefits	Potential Shortcomings
Reduces total waste sent to landfill.	Establishing recycling partnerships, new waste separation infrastructure and tracking systems can require significant upfront investment.
Encourages a circular approach to used oil and service materials.	Managing multiple waste streams and coordinating with external recycling or re-refining partners can add logistical complexity.
Creates opportunity for cost savings or additional value streams.	The system relies heavily on employees correctly sorting and tracking waste, which requires continuous training and oversight.

**Table 6:** Functional benefits and potential shortcomings of Concept 3 (spider-web-inspired precision signal detection).

Functional Benefits	Potential Shortcomings
Greater diagnostic accuracy through improved signal filtering.	Developing or acquiring advanced, context-aware diagnostic systems and adaptive dashboards can be expensive and IT infrastructure-intensive.
Technicians develop deeper fault-recognition skills through data-supported learning.	Effectiveness relies heavily on accurate, consistent technician data entry; incomplete data could compromise system learning.
Reduction in misdiagnoses and customer callbacks.	Technicians may initially resist new tools or find them complex, slowing early implementation.

**Table 7:** Functional requirements of AntLink with corresponding feature implementation and biomimicry alignment inspired by ant colony principles.

Functional Requirement	AntLink Feature Implementation	Biomimicry Alignment (Ant Colony Principle)
FR1: Accurate fault diagnosis	Shared, standardised intake form completed jointly by service advisor and technician; “pheromone trail” fault log.	Ants communicate locally via pheromone trails, ensuring fast, accurate information sharing → mirrors joint input and shared diagnostic updates.
FR2: Consistent repair quality	Standard quality checklist built into the app before job completion.	Ants follow consistent behavioural patterns that ensure reliable outcomes → standardised checklists ensure uniform repair quality.
FR3: Increase process standardisation	Standardised digital forms for intake, diagnosis and service records.	Ant colonies use repeatable structured behaviours → digital forms enforce uniform processes.
FR4: Effective communication of service issues	Shared, standardised issue-reports dashboard.	Ants signal changes immediately to the colony → shared dashboards keep service teams aligned in real time.
FR5: Technician capability development	Column chart of error type vs. frequency highlighting training needs.	Ants learn adaptively and switch roles based on need → chart analysis guides technicians to strengthen weak areas.
FR6: Reduce waste volume and improve sorting	Job resource report dashboard records resources used per job and disposal method (landfilled, recycled, reused, external contractor).	Ant colonies reuse resources and adapt resource flows to scarcity → tracking usage and disposal mirrors efficient, circular use of materials.
FR7: Encourage a sustainability mindset	Job resource report dashboard and column chart provide visibility and encourage sustainable choices.	Ant colonies sustain the whole ecosystem through collective resource management → resource transparency motivates collective responsibility.

**Table 8:** Survey statement descriptive statistics.

No.	Statement	Mean	SD
0	Please rate the degree to which you agree or disagree with the given problem statement.	3.833	1.169
1	AntLink’s shared intake and diagnostic forms will improve fault diagnosis accuracy.	3.500	1.517
2	The built-in quality checklist will help maintain consistent repair standards.	4.333	0.516
3	AntLink’s standardized digital intake, diagnostic and service records will increase process standardisation and efficiency.	3.667	1.506
4	The shared issues-report dashboard will improve effective communication of service issues.	4.167	0.753
5	The dashboards will help identify training needs and improve technician skills.	4.167	0.753
6	Tracking environmental KPIs will help reduce waste and improve resource sorting.	4.000	0.894
7	AntLink will encourage a stronger sustainability mindset across teams.	4.000	0.894
	Grand Mean/Mean SD	3.958	1.000

**Table 9:** Comparison of AntLink against Life’s Principles.

Life’s Principle	Relevant Sub-Principles	Justification (How AntLink Aligns)
Evolve to survive	Replicate Strategies that Work; Integrate the Unexpected; Reshuffle Information	AntLink mimics ant colony communication via a digital pheromone trail; a shared issue-reports dashboard lets unexpected changes be quickly shared and acted on; error-type and disposal-method column charts reorganise data to spot patterns.
Adapt to changing conditions	Maintain Integrity Through Self-Renewal; Incorporate Diversity; Embody Resilience	Employees keep the system accurate by updating information after each job; managers, service advisors and technicians all interact with the same system, adding diverse input; reduced reliance on a single role keeps operations running if one part fails.
Be locally attuned and responsive	Use Readily Available Materials and Energy; Cultivate Cooperative Relationships; Leverage Cyclic Processes; Use Feedback Loops	Dropdown and lookup lists reuse existing job, resource, employee and customer information; shared dashboards encourage cross-role collaboration; resource reporting tracks disposal methods to close material loops; issue reports and quality checklists provide feedback that improves future jobs.
Integrate development with growth	Self-Organise; Build from the Bottom-Up; Combine Modular and Nestled Components	Technicians and service advisors can input and access information directly without waiting for approvals; the design began with root cause analysis and grew into dashboards addressing each need; each dashboard handles a specific task while connecting into the larger system.
Be resourceful with material and energy	Fit Form to Function; Recycle All Materials; Use Low-Energy Processes; Use Multi-Functional Design	Digital forms, dashboards and checklists meet exact operational needs without added complexity; the job resource report dashboard encourages recycling and tracks waste practices; being digital reduces paper use and manual effort; dashboards serve multiple purposes such as recording data, guiding processes and training staff.
Use life-friendly chemistry	Employ Elegant Processes; Use a Small Subset of Elements	AntLink simplifies workflows through clear, step-by-step dashboards using a limited set of standardised digital tools (lookups, dropdowns, checklists) for efficiency; other sub-principles (chemistry in water; breaking down into benign constituents) were not applicable to a digital system.

## Data Availability

The data supporting the findings of this study are available from the corresponding author upon reasonable request, subject to the confidentiality agreement with the case-study dealership.

## Abbreviations

The following abbreviations are used in this manuscript:

**AHP**	Analytic Hierarchy Process
**BPM**	Business Process Map(ping)
**CEM**	Customer Experience Management
**ERD**	Entity-Relationship Diagram
**FR**	Functional Requirement
**GDP**	Gross Domestic Product
**ISIC**	International Standard Industrial Classification
**KPI**	Key Performance Indicator
**NEMWA**	National Environmental Management Waste Act
